# π-Electron reorganization in polycyclic aromatic hydrocarbons toward BT.2020-compliant narrowband red emitters

**DOI:** 10.1093/nsr/nwag219

**Published:** 2026-04-09

**Authors:** Jian Li, Pingxi Li, Ya Wang, Jiangliang Yin, Zhengyang Bin, Jingsong You

**Affiliations:** Key Laboratory of Green Chemistry and Technology of Ministry of Education, College of Chemistry, Sichuan University, Chengdu 610064, China; Key Laboratory of Green Chemistry and Technology of Ministry of Education, College of Chemistry, Sichuan University, Chengdu 610064, China; Key Laboratory of Green Chemistry and Technology of Ministry of Education, College of Chemistry, Sichuan University, Chengdu 610064, China; Collaborative Innovation Center of Materials Science, Nankai University, Tianjin 300350, China; Key Laboratory of Green Chemistry and Technology of Ministry of Education, College of Chemistry, Sichuan University, Chengdu 610064, China; Key Laboratory of Green Chemistry and Technology of Ministry of Education, College of Chemistry, Sichuan University, Chengdu 610064, China

**Keywords:** organic light-emitting diode, polycyclic aromatic hydrocarbons, BT.2020 red standard, skeletal reconstruction, π-electronic reorganization

## Abstract

Narrowband red emission remains a long-sought target for organic light-emitting diodes (OLEDs) but has proven exceptionally difficult to realize using polycyclic aromatic hydrocarbons (PAHs). Herein, we introduce a general molecular design paradigm that harnesses π-electron reorganization through skeletal reconstruction in PAHs. This topological engineering localizes aromaticity into discrete segments, intrinsically suppressing vibronic coupling and yielding exceptionally narrowband emission. Using ovalene as a model system, we created a family of PAH-based fluorophores spanning yellow to deep red (559–670 nm) with exceptionally narrow full-width at half-maximum (FWHM) values of 18–24 nm. When integrated into sensitized OLEDs, these emitters deliver record-setting device performance. A representative compound produces sharp red emission at 639 nm with a FWHM of only 28 nm/0.085 eV, Commission Internationale de l’Éclairage (CIE) coordinates of [0.704, 0.294] that precisely match the BT.2020 red standard ([0.708, 0.292]), and an external quantum efficiency exceeding 24%—the highest reported value for traditional red fluorescent systems. To our knowledge, this constitutes the first demonstration of a BT.2020-compliant red emitter with FWHM below 30 nm.

## Teaser

This work develops a high color purity PAH-based fluorophores design platform *via* a π-electron reorganization strategy, allowing the precise synthesis of intrinsic narrowband emitters for high-performance BT.2020 standard red OLEDs.

## INTRODUCTION

Since their inception in 1987 [1], organic light-emitting diodes (OLEDs) have evolved from a laboratory curiosity into a dominant platform for next-generation displays, steadily displacing conventional technologies in both consumer electronics and high-end visualization systems [2,3]. Fluorescent emitters, the earliest class adopted in OLED architectures, offer ease of synthesis and broad color versatility but fundamentally limit internal quantum efficiency (IQE) to 25% because triplet excitons cannot contribute to light emission [4,5]. This efficiency ceiling was subsequently surpassed by phosphorescent [6,7] and thermally activated delayed fluorescence (TADF) [8] emitters, which enable efficient triplet harvesting and near-unity IQE through strong spin–orbit coupling or reverse intersystem crossing. Despite these advances, both classes face persistent obstacles in meeting the stringent color purity required for ultra-high-definition (UHD) displays [9,10] conforming to BT.2020 standards [11]. Achieving high-fidelity color reproduction demands electroluminescence with a narrow full-width at half-maximum (FWHM) [12], yet most organic emitters generate broad spectra [9,10], forcing the use of external optical filters that incur optical losses and complicate device engineering [13,14]. Multiple-resonance thermally activated delayed fluorescence (MR-TADF) materials have emerged as a promising solution, achieving sharp emission bands by suppressing vibronic coupling through spatial separation of frontier orbitals within rigid, heteroatom-doped π-frameworks [15–17]. These emitters have delivered impressive efficiency and spectral precision in the blue and green regions [18–21], but translating this success to the red region has proven difficult [22]. Current MR-TADF red emitters typically display broad FWHMs exceeding 40 nm [22]—well above the threshold for high-fidelity color rendering—leaving this as a key challenge in OLED material development.

Recent progress in TADF-sensitized fluorescence and phosphorescence-sensitized fluorescence (PSF) OLEDs has revitalized interest in classical organic fluorophores by circumventing the intrinsic 25% IQE ceiling of purely fluorescent emitters [23–30]. The non-emissive triplet excitons generated within the emitting layers are efficiently harvested by the sensitizers and then transferred to the emissive singlet manifold of the fluorescent dopants, which ultimately enable high external quantum efficiency (EQE) [31–33]. Within this landscape, polycyclic aromatic hydrocarbons (PAHs) have gained prominence as sensitized OLED emitters [32] owing to their intrinsically rigid π-conjugated frameworks, structural tunability, and remarkable photostability [34,35]. Furthermore, the intrinsically non-polar backbone of all-carbon PAHs renders their frontier molecular orbitals (FMOs) less susceptible to perturbation by local dielectric fields. Consequently, both the emission peak and spectral bandwidth of PAHs remain largely invariant when integrated with polar sensitizers or host materials, which effectively minimizes spectral broadening and shifting [36,37]. Representative emitters such as 2,5,8,11-tetra-*tert*-butylperylene, 2,8-di-*tert*-butyl-5,11-di(4-*tert*-butylphenyl)-6,12-diphenyltetracene, and 5,10,15,20-tetraphenyl-benzo[5,6]indeno[1,2,3-*cd*]benzo[5,6]indeno[1,2,3-*lm*]perylene exhibit excellent performance when paired with suitable sensitizers [32]. Nevertheless, most PAHs intrinsically exhibit broad emission bands and inadequate spectral coverage in the red region [38], restricting their suitability for color-critical display technologies that demand both high efficiency and narrowband emission. These challenges have driven a renewed push toward the rational design of red-emitting PAHs featuring spectrally sharp and long-wavelength emission.

**Figure 1.**
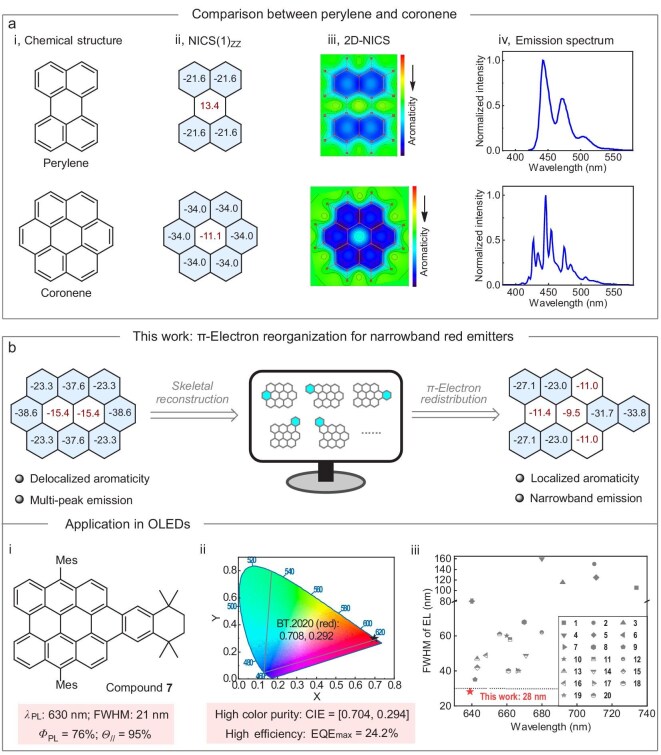
Molecular design concept. (a) Comparison between perylene and coronene. (i) Molecular structures; (ii) nuclear independent chemical shift (NICS) values; (iii) 2D-NICS maps; and (iv) emission spectrum in toluene solution (1.0 × 10^−5^ M). (b) This work: π-electron reorganization for narrowband red emitters. For application in OLEDs: (i) structure and photophysical properties of representative compound **7**; (ii) performance of the OLED device based on compound **7**; and (iii) summary of emitters approaching or meeting BT.2020 red standard in OLED devices. Details of the reported FWHM values are summarized in Table S4, while additional comparative references are provided in the Supplementary data.

Our recent investigations, integrating electronic-structure calculations with comprehensive photophysical characterization, reveal that the emission bandwidth of PAHs is strongly correlated with both the degree and topology of aromatic localization [39]. The localized aromaticity in these π-segments effectively suppresses vibrational coupling and excited-state structural relaxation—key contributors to spectral broadening and shoulder formation [39]. This raises a fundamental design question: what extent and arrangement of localized aromaticity is required to achieve intrinsically narrow emission? Comparative analysis of prototypical examples provides instructive contrasts: coronene exhibits extensive π-delocalization across its entire framework, producing vibrationally broadened emission, whereas perylene confines delocalization to two naphthalene-like segments, resulting in fewer vibronic features and narrower spectra (Fig. 1a) [40,41]. These observations point toward a generalizable design principle: disrupting global delocalization while maximizing the number of independently localized aromatic segments enhances spectral purity. From a topological perspective, PAHs can be viewed as assemblies of fused benzene units arranged in linear, angular, or branched motifs, each dictating distinct electronic delocalization landscapes [42]. Even subtle variations in fusion topology, as exemplified by the contrasting excited-state behaviors of anthracene and phenanthrene, underscore the sensitivity of photophysical properties to molecular geometry [43]. These insights indicate that systematic skeletal reconfiguration provides a rational strategy to enforce aromatic localization, tailor emission bandwidth, and optimize frontier orbital alignment (Fig. 1b). Analogous to assembling LEGO (the Danish toy brand famous for its interlocking plastic bricks) bricks, such modular design allows for the programmable construction of hydrocarbon narrowband emitters, offering not only practical benefits for high-definition OLEDs but also deeper insight into the electronic structure–property relationships in excited-state conjugated systems.

## RESULTS AND DISCUSSION

**Figure 2.**
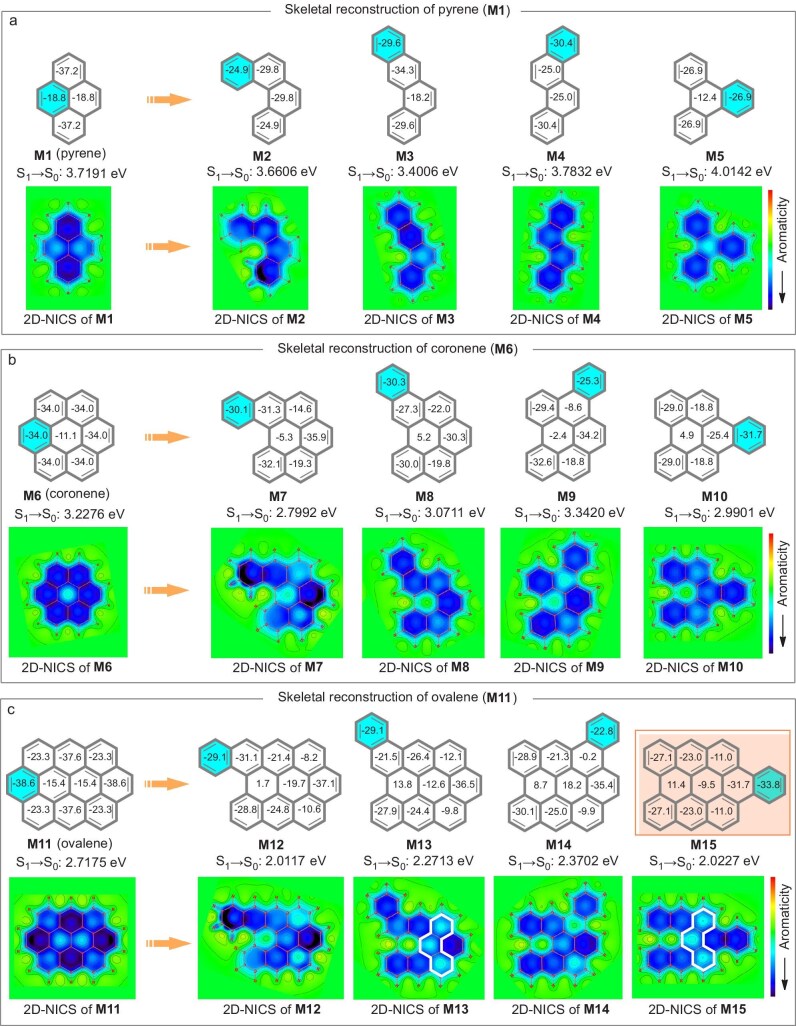
Proof of concept: π-electron reorganization induced by skeletal reconstruction. (a) Skeletal reconstruction of pyrene. (b) Skeletal reconstruction of coronene. (c) Skeletal reconstruction of ovalene. The NICS value, 2D-NICS and S_1_→S_0_ energy level calculations of molecules were calculated at the B3LYP/6-31G(d) level of theory.

**Figure 3.**
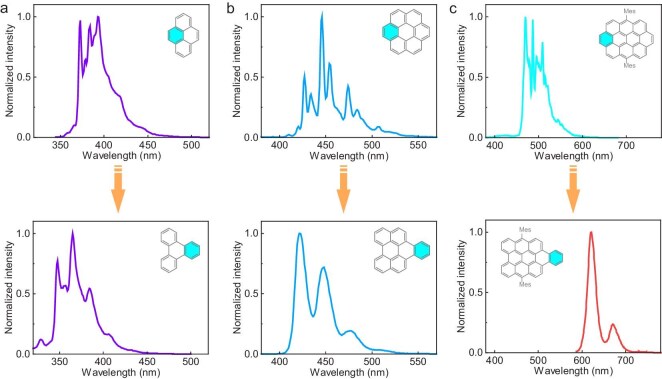
The emission spectral changes induced by π-electron reorganization. (a) Emission spectra of **M1** and **M5**. (b) Emission spectra of **M6** and **M10**. (c) Emission spectra of **1** and **2**. The spectra were collected in toluene solution (1.0 × 10^−5^ M).

As a proof of concept, we employed the nuclear independent chemical shift (NICS) as the primary metric for aromaticity [44], supplemented by 2D-NICS mapping to visualize, in a spatially resolved manner, the π-electron reorganization induced by skeletal reconstruction [45]. Pyrene, a prototypical tetracyclic aromatic hydrocarbon widely used in organic optoelectronics [46], was selected as the starting framework owing to its stereoelectronic resemblance to coronene, which reflects a globally delocalized aromatic character [47]. Drawing inspiration from both coronene and perylene, we implemented a ‘disassembly-relocation’ strategy, in which one central ring of pyrene is excised and repositioned elsewhere in the carbon framework (Figs 2a and S1). This rearrangement yields a conjugation-extended tetracene analogue, where NICS analysis indicates substantial reorganization of the π-electron network. This approach is particularly illuminating in the case of **M5** (triphenylene), generated by annulating a benzene ring onto the 9,10-positions of phenanthrene. Compared with **M2**–**M4, M5** displays markedly enhanced localization of aromaticity, underscoring the pronounced sensitivity of π-electron distribution to subtle topological modifications. Consistent with this electronic reorganization, photophysical measurements revealed a moderate attenuation of the shoulder-band intensity in the emission spectrum of **M5** relative to **M1** (Fig. 3a).

The impact of skeletal reconstruction becomes more pronounced in larger polycyclic systems such as coronene, which, like pyrene, can be theoretically reshaped into derivatives **M7**–**M10** based on the benzo[*ghi*]perylene scaffold (Figs 2b and S2). Computations indicate that excising a peripheral ring reduces the aromaticity of the central core, while relocating the removed benzene unit to the molecular periphery induces distinct patterns of aromaticity localization. The contrast between **M8** and **M9** is particularly striking: despite sharing the same carbon framework, their π-electron delocalization pathways differ profoundly, with **M8** sustaining a far more extended conjugation network. Such topology-driven reorganization of π electrons directly influences photophysical behavior. Notably, **M10** exhibits a cleaner emission profile than coronene (**M6**) (Fig. 3b), and the reconstructed scaffolds display substantial variations in highest occupied molecular orbital (HOMO)–lowest unoccupied molecular orbital (LUMO) energy gaps, enabling wavelength-tunable emission. Although these gaps remain within the short-wavelength regime, the results highlight skeletal reconstruction as a versatile approach for simultaneously modulating aromaticity distribution and tailoring optical response in PAHs. To engineer a framework capable of narrow red emission, we reconfigured ovalene (**M11**) to expand its π-conjugated network (Figs 2c and S3). While the 10-ring topology permits up to 16 025 theoretical permutations, orders of magnitude greater than the 195 possible for the 7-ring system [48], we adopted reconstruction modes analogous to those applied to pyrene and coronene, as these are known to disrupt global aromaticity to varying degrees. Quantum-chemical analysis of the resulting derivatives (**M12**–**M15**) reveals pronounced π-electron reorganization and distinct patterns of localized aromaticity. While their electronic rearrangements parallel those observed in **M7**–**M10**, the ovalene congeners exhibit stronger aromatic-domain segmentation. In particular, **M13** and **M15** develop a weakly aromatic strip reminiscent of phenanthrene, accompanied by a longitudinal ‘fissure’ that increases the number of localized aromatic regions relative to **M8** and **M10**. Notably, **M12** and **M15** display calculated S_1_→S_0_ energy levels near 2.0 eV, consistent with emission wavelengths around 620 nm. Given its extensively segmented aromatic domains and a relatively symmetric architecture favorable for synthesis, **M15** was selected as the core framework for subsequent synthesis and photophysical evaluation.

The extended π-conjugation of the **M15** framework promotes exciton delocalization and non-radiative decay pathways, thereby suppressing emission and reducing the photoluminescence quantum yield ($\Phi_{\rm PL}$). At the same time, the increased molecular planarity lowers solubility, presenting challenges for purification and downstream material processing. To alleviate these drawbacks, sterically demanding mesityl (Mes) groups were introduced at the molecular periphery. The Mes-functionalized **M15** derivative (compound **2**) was synthesized through a Diels–Alder cycloaddition between 7,14-dimesitylphenanthro[1,10,9,8-*opqra*]perylene (**S1**) and anthranilic acid, which served as an *in situ* benzyne precursor. Photophysical measurements in toluene revealed a sharp emission maximum at 621 nm with an FWHM of 21 nm and a high $\Phi_{\rm PL}$ of 71%, accompanied by only a weak spectral shoulder (Fig. 3c). In contrast, the reference compound **1** displayed a broad, multi-peak emission profile (Fig. 3c).

In addition to examining the aromaticity variations of compounds **1** and **2**, frontier molecular orbitals (FMOs), reorganization energy (λ), and Huang–Rhys factor were investigated to further elucidate the origin of spectral narrowing. As shown in Fig. S4a, the HOMO and LUMO of both compounds are delocalized across the molecular backbone and display distinct spatial separation. However, the calculated reorganization energies of these compounds differ significantly (Fig. S4b). The lower reorganization energy of compound **2** (0.180 eV) compared to that of compound **1** (0.207 eV) reveals the suppressed structural relaxation upon emission and thus the potentially narrower FWHM in emission. Furthermore, the Huang–Rhys factor for the S_1_→S_0_ transition of the two molecules was also calculated and analyzed. The results shown in Fig. S4c reveal that compound **2** exhibits significantly weaker vibrational excitation in the high-frequency region (1300–1800 cm^−1^) relative to compound **1**, indicating inhibited stretching and scissoring modes of aromatic rings and thus potentially suppressed emission shoulder peaks. These findings indicate that π-electron reorganization arising from framework reconstruction effectively disrupts π-delocalization circuits and localizes aromaticity, suppressing vibrational coupling and structural relaxation, thereby affording intrinsically narrowband emission.

**Figure 4.**
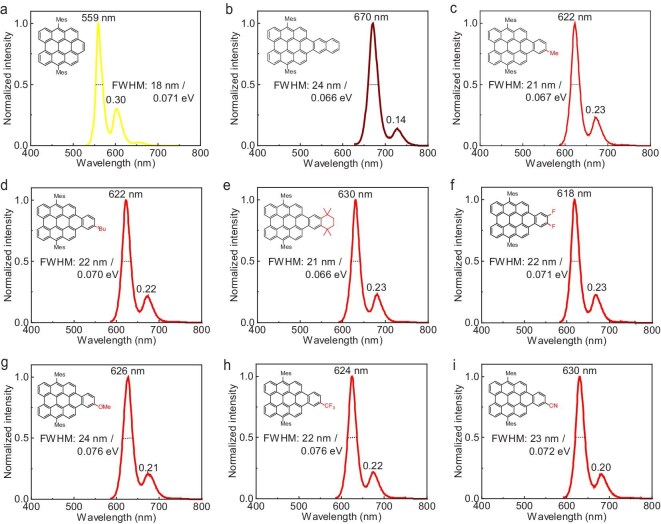
Emission spectra of the derivatives of compound **2**: (a) **3**, (b) **4**, (c) **5**, (d) **6**, (e) **7**, (f) **8**, (g) **9**, (h) **10** and (i) **11**. The spectra were collected in toluene solutions (1.0 × 10^−5^ M).

To further validate the robustness of this molecular design platform, we systematically modulated the π-conjugation length of the **M15** framework to shift the emission while preserving its narrow bandwidth. 2D-NICS calculations revealed that a weakly aromatic, phenanthrene-like strip along the longitudinal axis electronically decouples the π-systems on either side (Fig. 2c). Guided by this insight, substituting the ‘detached’ phenylene segment of compound **2** with either an ethylene or naphthalene moiety was expected to induce blue- or red-shifted emission, respectively, without compromising spectral purity. This design principle was validated synthetically through Diels–Alder cycloadditions of ethynyl or naphthynyl precursors with 1,4-dimesitylphenanthro[1,10,9,8-*opqra*]perylene (**S1**), affording compounds **3** and **4**. Compound **3**, with reduced conjugation, displayed a sharp yellow band at 559 nm (FWHM = 18 nm) (Fig. 4a), whereas compound **4**, with extended conjugation, exhibited a red-shifted emission at 670 nm with only a slight broadening (FWHM = 24 nm) (Fig. 4b). Such spectral redshift or blueshift induced by the variation in π-conjugation is consistent with the calculated decrease or increase in the energy gap (*E*_g_) and S_1_ energy level, respectively (Fig. S6). The strong correlation between these photophysical signatures and the corresponding 2D-NICS profiles highlights how localized π-electrons enable predictable control over narrowband emission (Fig. S5).

To achieve precise spectral tuning, we synthesized a library of derivatives of compound **2** via Diels–Alder cycloaddition, enabling systematic modulation of the emission properties (Table S1). All derivatives exhibited intense red fluorescence with peak wavelengths of 618–630 nm and FWHM values below 25 nm (Fig. 4 and Table S2). Substitution patterns exerted distinct electronic and steric effects. Alkyl groups induced bathochromic shifts, with the cycloalkyl analogue **7** redshifted to 630 nm while steric congestion concurrently suppressed non-radiative decay, enhancing $\Phi_{\rm PL}$ to 76% (Fig. 4e). Fluorinated derivatives produced hypsochromic shifts yet delivered the highest $\Phi_{\rm PL}$ value in the series (82%) (Fig. 4f). Relative to compound **2**, electron-rich methoxy substitution in compound **9** induced a redshift accompanied by the broadest emission profile (FWHM = 24 nm) (Fig. 4g), while electron-withdrawing groups such as trifluoromethyl and cyano (compounds **10** and **11**) also yielded redshifted but spectrally broadened emissions (Fig. 4h and i). As calculated, the introduction of these substituents exerts little influence on the topological distributions of the HOMO and LUMO of the backbone but induces subtle variations in the S_1_ energy level and the energy gap (*E*_g_). This consequently leads to shifts in the emission peaks while maintaining similarly narrow FWHM values (Fig. S6). Collectively, these results identify compounds **2** and **7** as optimal molecular scaffolds that combine narrowband emission with high efficiency, providing a robust basis for integration into red-emitting OLED devices (Table 1 and Supplementary data for device-level data). Prior to device fabrication and characterization, the solvatochromic behavior of compounds **2** and **7** was further investigated, and their emission characteristics in the solid state were examined in several widely employed host matrices. Owing to the small dipole moment associated with their all-carbon skeleton, compounds **2** and **7** exhibit similar absorption and emission profiles in various solvents, with negligible variations in both peak position and FWHM (Fig. S10). In the solid state, neither compound exhibits a significant shift in emission peak maximum nor a remarkable change in FWHM upon doping into three structurally and electronically distinct host matrices: the electron-deficient host bis[2-(diphenylphosphino)phenyl]ether oxide, the electron-rich host 3,3′-di(9*H*-carbazol-9-yl)-1,1′-biphenyl, or the TADF-type host 1,3-dihydro-1,1-dimethyl-3-(3-(4,6-diphenyl-1,3,5-triazin-2-yl)phenyl)indeno[2,1-*b*]carbazole (DMIC-TRZ) (Fig. S11). These observations further validate the intrinsic advantages of the rigid all-carbon skeletons for the application as emitters in OLED devices. The electrochemical properties and thermal robustness of compounds **2** and **7** were subsequently examined to assess their suitability for OLED applications. Cyclic voltammetry measurements revealed nearly identical redox potentials, corresponding to HOMO–LUMO energy levels of −4.91/−3.01 and −4.87/−2.99 eV, respectively (Fig. S13). Thermogravimetric analysis (TGA) further showed exceptional stability, with decomposition temperatures (corresponding to 95% weight loss) of 430 and 434°C (Fig. S14), ensuring compatibility with vacuum deposition protocols commonly employed in device fabrication.

**Table 1.** Summary of OLED performance.

Emitter	*V*_on_^a^ (V)	*λ*_EL_^b^ (nm)	CIE^b^ (*x, y*)	FWHM^b^ (nm)/(eV)	EQE_max/1000/10 000_ (%)	PE_max_ (lm W^−1^)	$\Phi$ **_//_** ^c^
**2**	2.7	634, 691 (0.23^d^)	0.698, 0.298	26/0.070	20.2/17.4/14.8	16.2	0.92
**7**	2.8	639, 695 (0.27^d^)	0.704, 0.294	28/0.085	24.2/20.0/16.7	15.0	0.95

a Turn-on voltage at 1 cd m^–2^. ^b^Measured at 1000 cd m^–2^. ^c^Measured in 0.5 and 0.8 wt% DMIC-TRZ host-blended film, respectively. ^d^The relative intensity of the shoulder peak.

**Figure 5.**
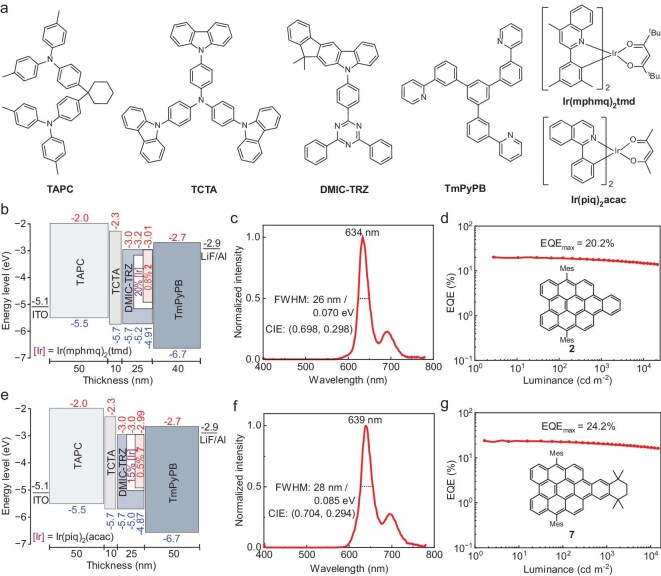
Electroluminescence (EL) performance. (a) Chemical structures of the materials employed in each device. (b) Device architecture and energy-level diagram of the functional materials for OLEDs based on **2**. (c) EL spectra and CIE coordinates of **2**. (d) External quantum efficiency–luminance curve of the device and chemical structure of **2**. (e) Device architecture and energy-level diagram of the functional materials for OLEDs based on **7**. (f) EL spectra and CIE coordinates of **7**. (g) External quantum efficiency–luminance curve of the device and chemical structure of **7**.

To evaluate the electroluminescence properties of emitter **2** within a PSF-OLED framework, we fabricated optimized devices with the configuration of indium tin oxide/1,1-bis((di-4-tolylamino)phenyl)-cyclohexane (TAPC) (50 nm)/tris(4-carbazolyl-9-ylphenyl)amine (TCTA) (10 nm)/emitting layer (EML) (25 nm)/1,3,5-tri(*m*-pyridin-3-ylphenyl)benzene (TmPyPB) (40 nm)/LiF (0.8 nm)/Al (100 nm). The EML comprised a ternary system of DMIC-TRZ doped with 20 wt% Ir(mphmq)_2_tmd and 0.8 wt% emitter **2**. In this architecture, TAPC and TmPyPB function as hole- and electron-transport layers, respectively, TCTA serves as a hole-blocking layer, and LiF facilitates electron injection (molecular structures shown in Fig. 5a; energy level alignment in Fig. 5b). Given the significant spectral overlap between the absorption of the emitter and the emission of the red iridium complex, which is favorable for efficient energy transfer, Ir(mphmq)_2_tmd was employed as a sensitizer to promote triplet exciton utilization in the PSF-OLEDs (Fig. S12a). The resulting OLEDs exhibit saturated red emission with a peak at 634 nm, a narrow FWHM of 26 nm/0.070 eV, and Commission Internationale de l’Éclairage (CIE) chromaticity coordinates of [0.698, 0.298] (Fig. 5c and Table 1). The emission spectrum is consistent with that of the device without using a phosphorescence sensitizer, indicating the efficient energy transfer in the PSF-OLEDs (Fig. S18). Owing to the high horizontal transition dipole ratio ($\Phi$_//_ = 0.92) imparted by the rigid, planar π-conjugated framework, the devices deliver an external quantum efficiency (EQE_max_) of 20.2% (Fig. 5d and Table 1). Even at a luminance of 10 000 cd m^−2^, the EQE remains 14.8%, corresponding to a modest efficiency roll-off of only 25% under high-brightness operation (Table 1).

To suppress aggregation-caused quenching in the emitting layer, emitter **7** was designed with a sterically hindered cycloalkyl unit and subsequently employed as the emitter in device fabrication. Given its more pronounced bathochromic shift relative to emitter **2**, the optimized device adopted Ir(piq)_2_acac, a sensitizer with deeper-red emission than Ir(mphmq)_2_tmd, to ensure spectral matching between the phosphorescence sensitizer and the emitter (Figs 5e and S12b). This strategy yielded a significant improvement in performance: the emitter **7**-based OLED reached a record-high EQE_max_ of 24.2% based on traditional red fluorescent emitters (details of EQE values reported in the cited literature are summarized in Table S3), surpassing the 20.2% achieved by its emitter **2** counterpart (Fig. 5g). In addition, emission was subtly redshifted, peaking at 639 nm, with an ultra-small FWHM of 28 nm/0.085 eV and CIE chromaticity coordinates of [0.704, 0.294], aligned with the BT.2020 red standard ([0.708, 0.292]) (Fig. 5f and Table 1). Similar to the former, this electroluminescence spectrum is also consistent with that of the device without using a phosphorescence sensitizer, indicating efficient energy transfer in the PSF-OLEDs (Fig. S18). It is worth noting that this represents the first example of BT.2020-compliant red emitters with a narrow FWHM of less than 30 nm (Fig. 1b; details of FWHM values reported in the cited references are summarized in Table S4).

## CONCLUSION

In summary, we introduce a general strategy for realizing narrowband emission by precisely modulating π-electron delocalization through topological engineering of PAHs. Systematic skeletal reconstruction reorganizes π-electrons to disrupt π-delocalization circuits and thus form localized aromatic domains that effectively suppress vibronic coupling and enhance spectral purity. Guided by this principle, we synthesized a family of efficient PAH-based fluorophores that deliver sharp emission peaks spanning 559–670 nm with FWHM values as narrow as 18 nm. A representative emitter, compound **7**, delivers exceptional electroluminescence performance in sensitized OLED devices, reaching a high EQE_max_ of 24.2% along with the BT.2020 red color standard. Beyond providing a practical platform of high-purity narrowband emitters, these findings demonstrate that precise control of localized aromaticity is a broadly applicable strategy for discovering narrowband organic emitters.

## METHODS

The detailed preparation and characterization methods of materials are available as Supplementary data.

## Supplementary Material

nwag219_Supplemental_File
