# Supplementary material for: π-Electron reorganization in polycyclic aromatic hydrocarbons toward BT.2020-compliant narrowband red emitters
Source: Natl Sci Rev. 2026 Apr 9;13(10):nwag219. doi: 10.1093/nsr/nwag219 (PMC13251888; doi:10.1093/nsr/nwag219)
Supplement: nwag219_Supplemental_File [file nwag219_supplemental_file.pdf]

Supplementary Data

π-Electron reorganization in polycyclic aromatic hydrocarbons towards BT.2020-compliant narrowband red emitters

Jian Li[1], Pingxi Li[1], Ya Wang[1], Jiangliang Yin[2], Zhengyang Bin,[1,]* Jingsong You[1,]*

[1]Key Laboratory of Green Chemistry and Technology of Ministry of Education, College of Chemistry, Sichuan University, Chengdu 610064, China;

[2]Collaborative Innovation Center of Materials Science, Nankai University, Tianjin 300350, China.

*Corresponding author. E-mail: binzhengyang@scu.edu.cn; jsyou@scu.edu.cn

Contents

I. General Information 3

II. Theoretical Calculation 5

III. Synthesis of Compounds 10

IV. Photophysical Properties 18

V. Cyclic Voltammograms 23

VI. Thermogravimetric Analysis 23

VII. Characterization of OLED devices. 24

VIII. Performance Summary of the Reported Red Emitters 26

IX. Reference 29

X. Copies of NMR Spectra 32

I. General Information

1. Reagents, solvents, and reactions. All chemical reagents were purchased from commercial sources and were used without further purification unless stated otherwise. Solvents were purified and dried using an Innovative Technology PS-MD-5 Solvent Purification System. Substrate dimesitylphenanthro[1,10,9,8-*opqra*]perylene (**S1**) was synthesized according to literature known protocols[1]. The reactions and experiments sensitive to dioxygen were performed using Schlenk techniques and with nitrogen-saturated solvents. Prior to use all the glassware were dried in oven at 120 °C for 2 h.

2. Chromatography. TLC was performed on 0.20 mm HUANGHAI silica gel HSGF 254 plates and visualized under UV light (λ_{max} = 254 nm) or by staining with potassium permanganate ($KMnO_4$). Silica flash chromatography was performed on HAIYANG 100–200 mesh silica gel TP-3.

3. NMR Spectroscopy. NMR spectra were recorded on Varian Inova 400 MHz NMR spectrometer or Bruker Avance III-800 MHz NMR spectrometer. Chemical shifts are expressed in ppm relative to solvent signals: $CDCl_3$ (^{1}H, 7.26 ppm, ^{13}C, 77.16 ppm); coupling constants are expressed in Hz. NMR spectra were processed using MestReNova software (Ver. 14.0.0-23239).

4. High resolution mass spectrometry (HRMS). HRMS data were obtained with Shimadzu LCMS-IT-TOF spectrometer or Waters X500R spectrometer.

5. UV−vis and fluorescence spectroscopy. UV−vis spectra were collected on HITACHI U-2910 spectrometer in toluene (1.0×10^{-5} mol/L). Fluorescent spectra and photoluminescent quantum yield were collected on a Horiba Jobin Yvon-Edison Fluoromax-3 fluorescence spectrometer with a calibrated integrating sphere system in toluene (1.0×10^{-5} mol/L).

7. Transient photoluminescent decay spectroscopy. Transient photoluminescent decay spectra were procured with Horiba Single Photon Counting Controller: FluoroHub and Horiba TBX Picosecond Photon Detection.

8. Cyclic voltammograms (CV). CV measurement was performed on CHI660 with a solution of tetrabutylammonium hexafluorophosphate (Bu_4NPF_6) in dry DCM (0.1

mol/L) as electrolyte and ferrocene/ferrocenium (Fc/Fc^+) as internal standard under nitrogen atmosphere.

9. Thermogravimetric analysis (TGA). TGA was carried out using DTG-60(H) at a rate of 10 ºC/min under nitrogen atmosphere. The test temperature range is from 25 °C to 600 °C.

10. Theoretical calculation. All theoretical calculations were performed using Gaussian 09 serials software[2], using density functional theory (DFT) and time-dependent DFT (TD-DFT) method with the B3LYP functional with Grimme's D3 dispersion correction. Frequency analysis was performed to verify the stationary state geometry. GaussView (Ver. 5.0) software was used to generate graphical images of frontier molecular orbitals (FMOs). The magnetic shielding tensors were calculated using Gaussian 09, and the nucleus independent chemical shift (NICS) analysis was performed and visualized using Multiwfn (Ver. 3.8) software[3]. Considering the non-planarity of molecules, the $NICS(1)_{ZZ}$ values were obtained by placing dummy atom at 1 Å above and below each ring.

11. Organic light-emitting diode (OLED) devices fabrication and characterization. Indium-tin-oxide (ITO) coated glass with a sheet resistance of about 15 Ω sq^{-1} was used as the anode substrate. Ahead of film deposition, ITO substrates were cleaned with alkaline detergent, boiled deionized water, deionized water in ultrasonic bath, dried in an oven, and finally treated with oxygen plasma for 10.0 min to enhance the surface work function of ITO anode. All the organic layers, LiF and Al were deposited onto the ITO-coated substrates by thermal evaporation in a high vacuum chamber below 5.0×10^{-4} Pa in an inert gas glovebox. The doped and co-doped layers were prepared by co evaporating dopants and host materials from individual sources, and the doping concentrations were modulated by controlling the evaporation rates of dopants. Current density versus voltage and luminance versus voltage characteristics of OLEDs were measured by using KEYSIGHT B1500A. and model DLM-100Z photometer. The luminance and electroluminescence spectra were collected with model DLM-100Z photometer and OPT2000 spectrophotometer, respectively.

II. Theoretical Calculation

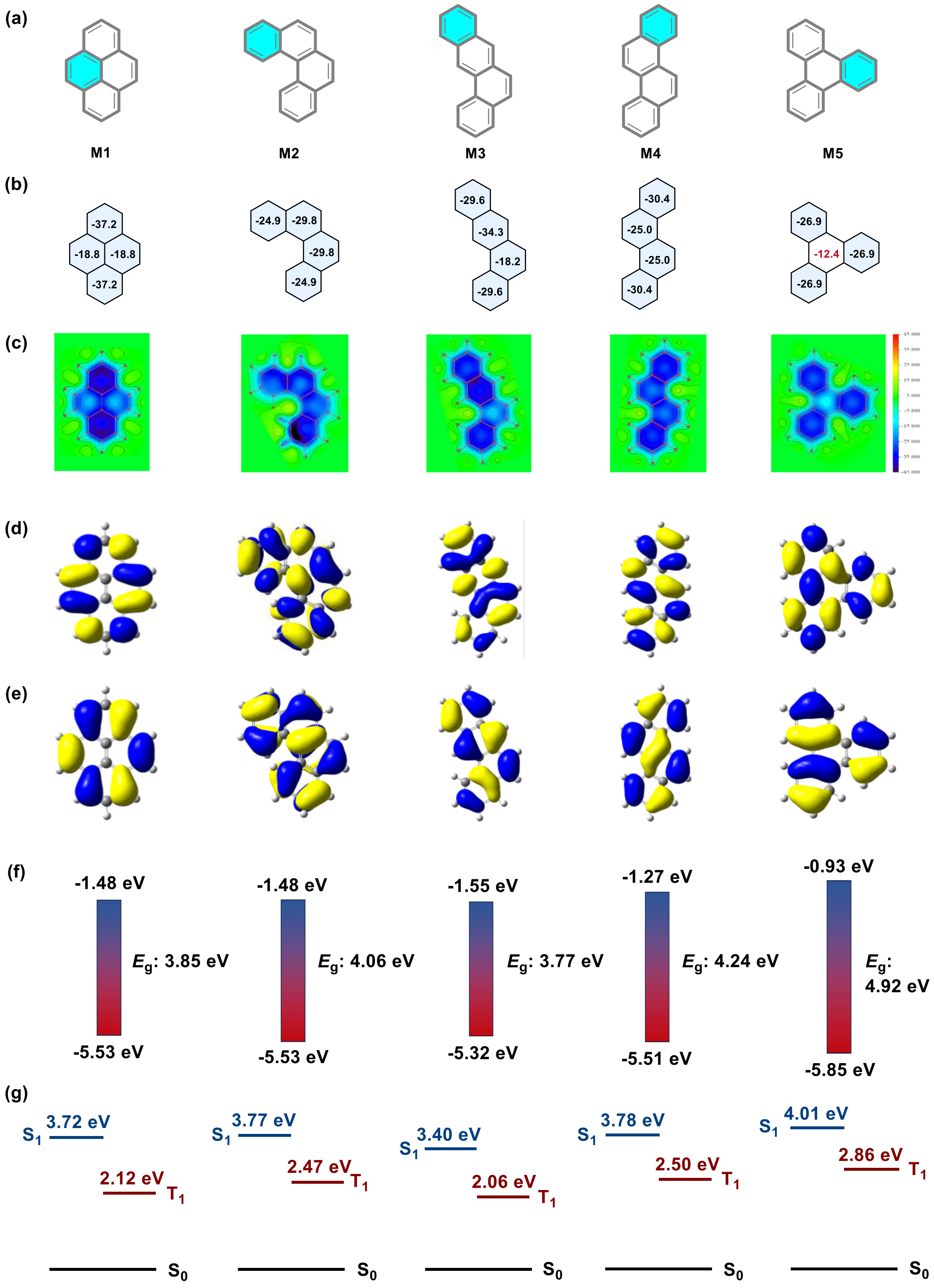

Figure S1. The theoretical calculations of **M1-M5**. (a) The structure of molecules (b) The NICS(1)$_{zz}$ values. (c) The 2D-NICS maps. (d) The visualized LUMOs. (e) The visualized HOMOs. (f) The energies of HOMO, LUMO, and HOMO-LUMO gap. (g) The energies of S_1 and T_1.

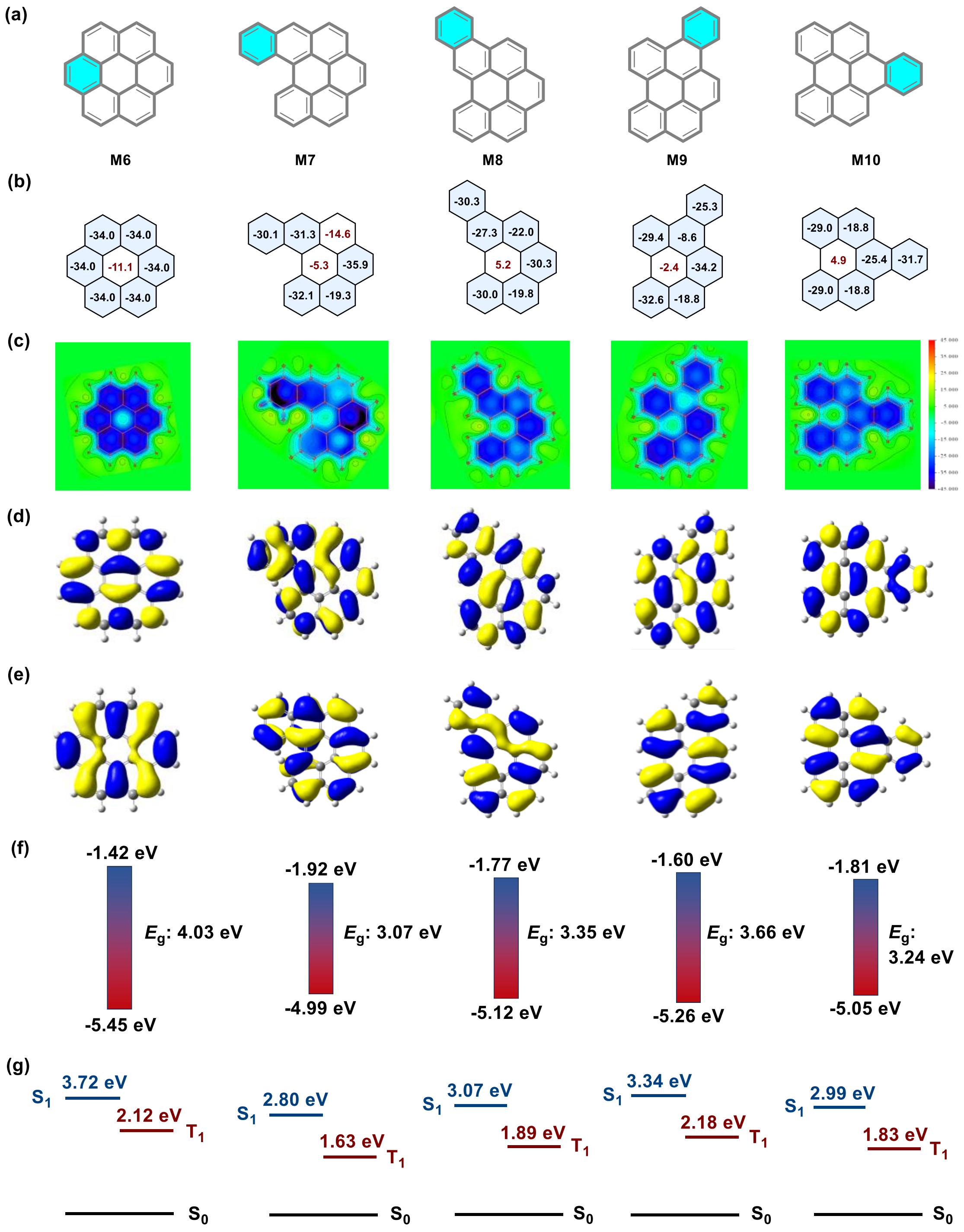

Figure S2. The theoretical calculations of **M6-M10**. (a) The structure of molecules (b) The NICS(1)$_{zz}$ values. (c) The 2D-NICS maps. (d) The visualized LUMOs. (e) The visualized HOMOs. (f) The energies of HOMO, LUMO, and HOMO-LUMO gap. (g) The energies of S_1 and T_1.

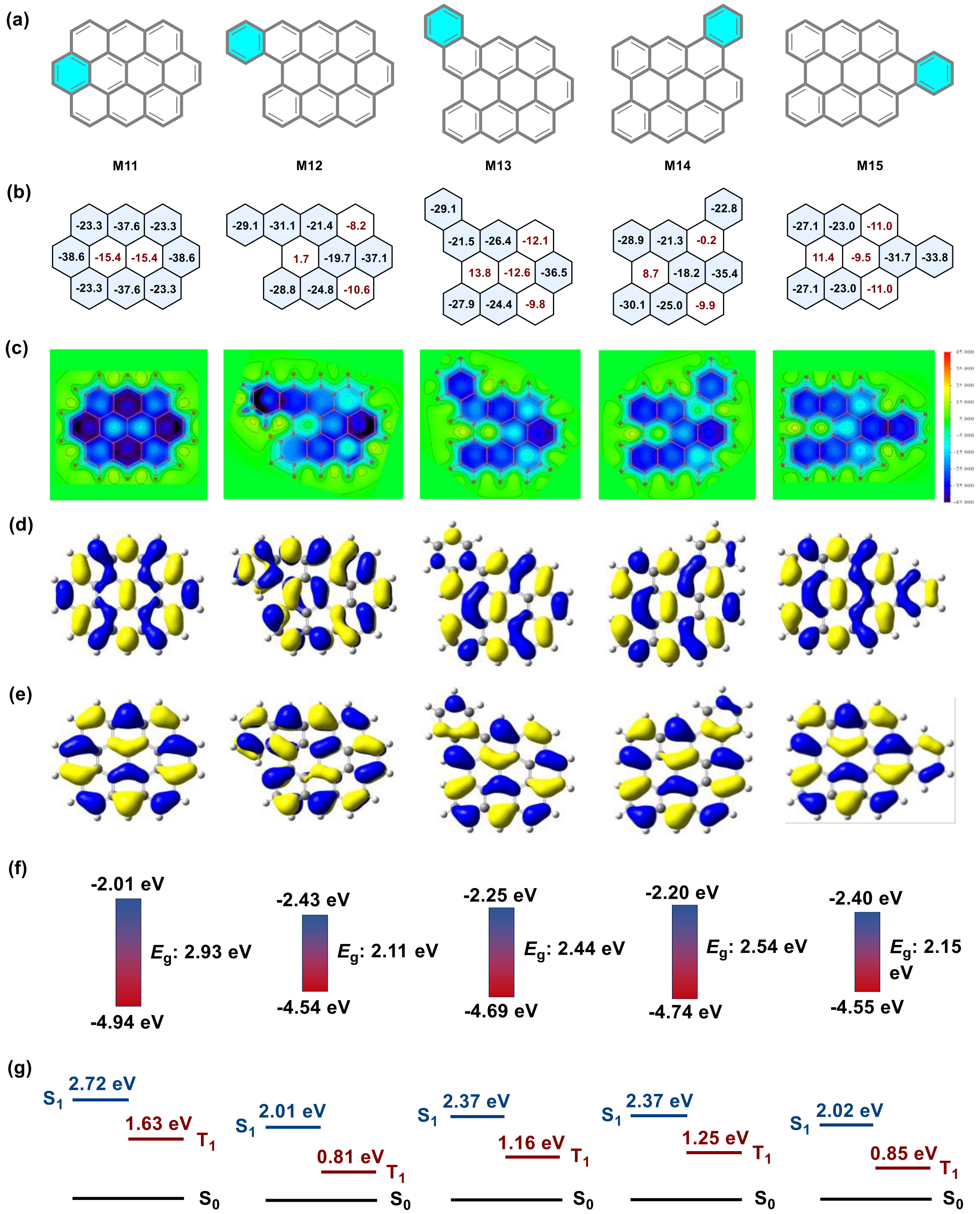

Figure S3. The theoretical calculations of **M11-M15**. (a) The structure of molecules (b) The NICS(1)$_{zz}$ values. (c) The 2D-NICS maps. (d) The visualized LUMOs. (e) The visualized HOMOs. (f) The energies of HOMO, LUMO, and HOMO-LUMO gap. (g) The energies of S_1 and T_1.

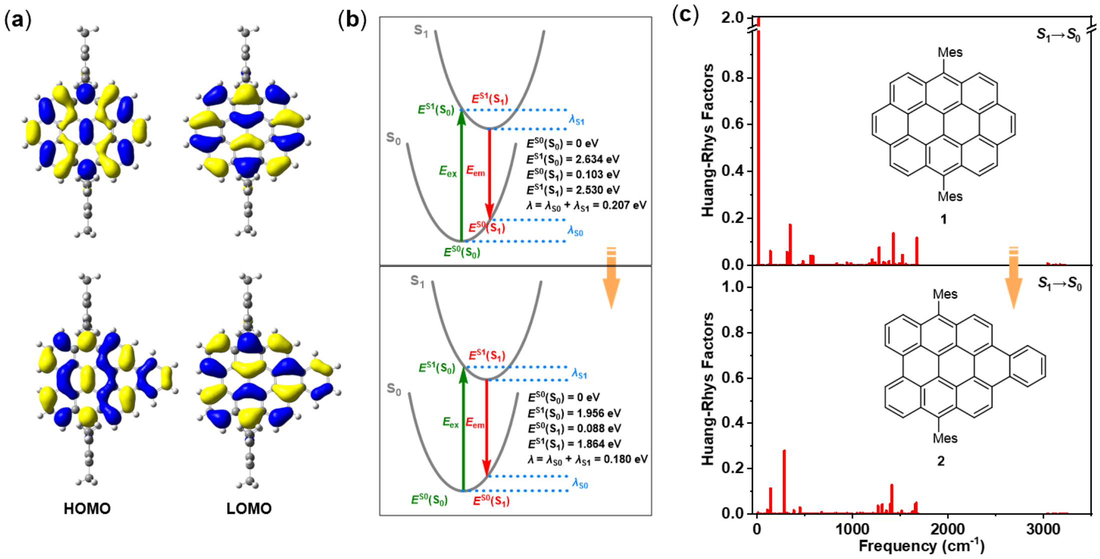

Figure S4. Theoretical calculations of compounds **1** and **2**. (a) Visualization of HOMO and LUMO orbitals. (b) Calculated Huang–Rhys factor for the $S_1 \rightarrow S_0$ transition. (c) Calculated reorganization energy (λ) for the $S_1 \rightarrow S_0$ transition.

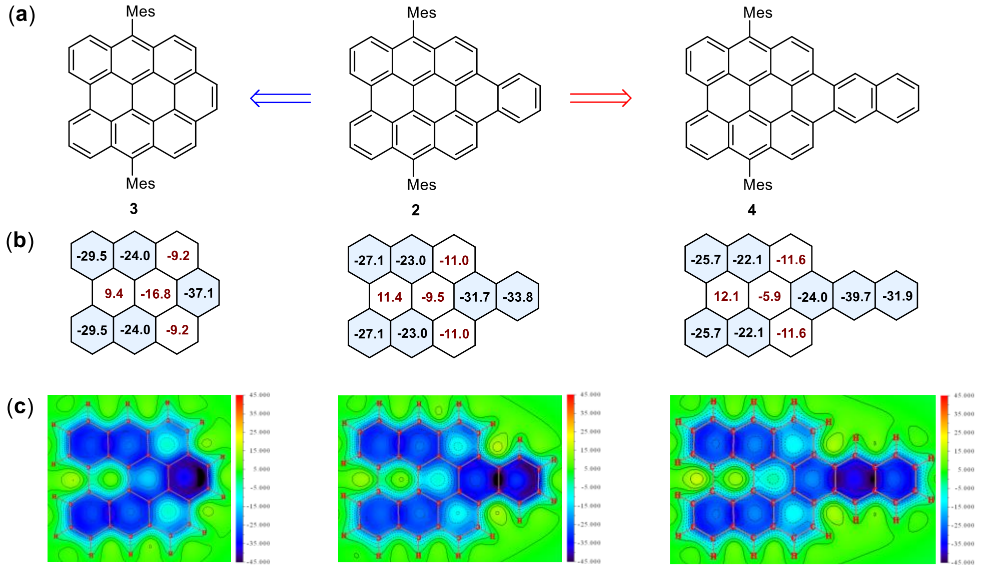

Figure S5. The aromaticity analysis of compounds **2-4**. (a) The structure of molecules (b) The NICS(1)$_{zz}$ values. (c) The 2D-NICS maps.

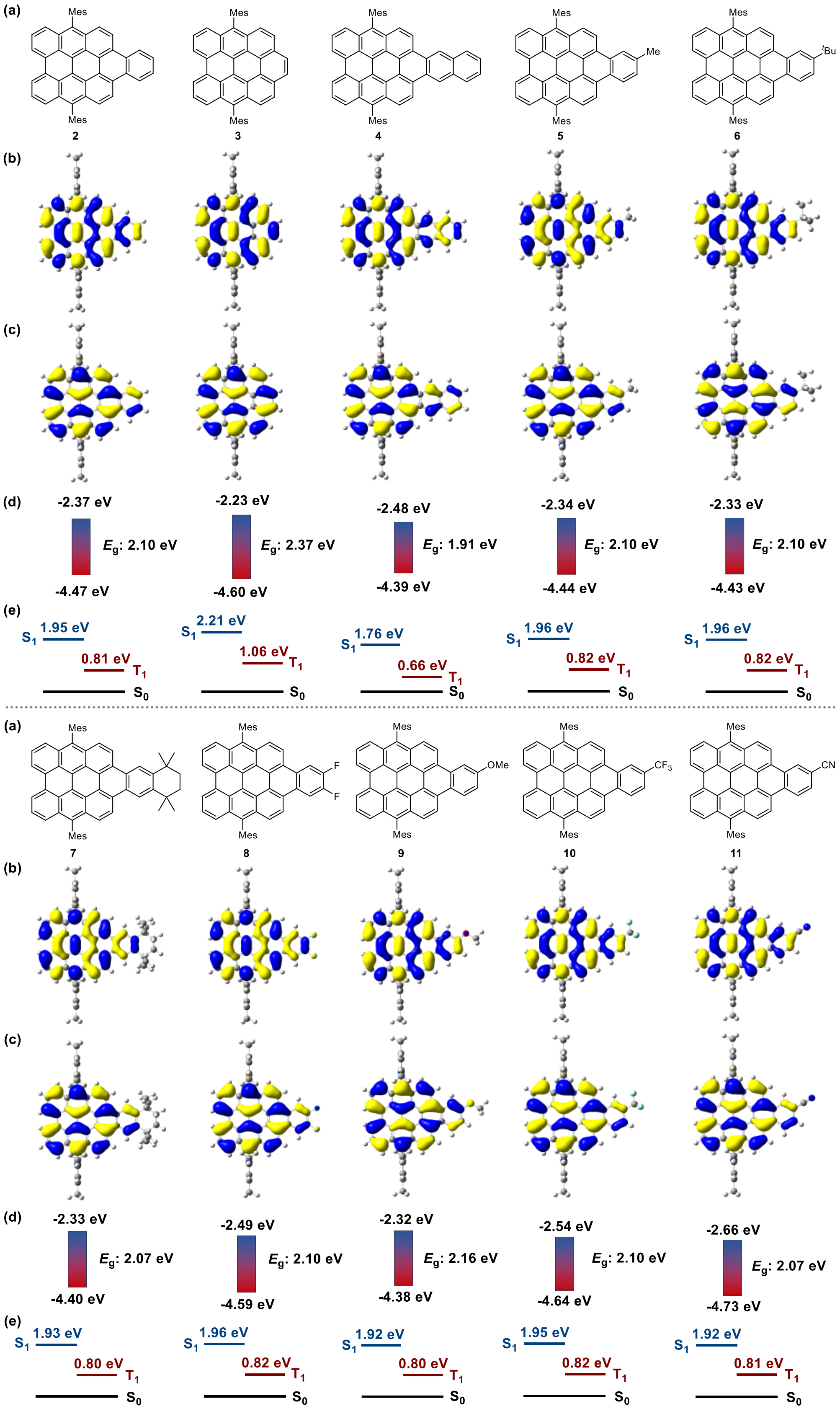

Figure S6. The theoretical calculations of **2-11**. (a) The structure of molecules. (b) The visualized LUMOs. (c) The visualized HOMOs. (d) The energies of HOMO, LUMO, and HOMO-LUMO gap. (e) The energies of S_1 and T_1.

III. Synthesis of Compounds

1. Synthesis of 7,14-dimesitylovalene (1)

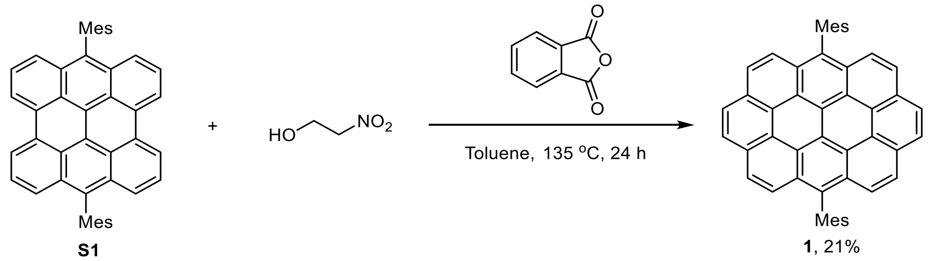

A 100 mL Schlenk tube with a magnetic stir bar was charged with 7,14-dimesitylphenanthro[1,10,9,8-*opqra*]perylene (**S1**) (50.2 mg, 0.085 mmol), 2-nitroethan-1-ol (300.0 µL, 4.25 mmol), and toluene (10.0 mL). Subsequently, isobenzofuran-1,3-dione (650.3 mg, 4.39 mmol) was added to the mixture at ambient temperature. The vessel was sealed tightly with a screw cap, then stirred at 135 °C in an oil bath for 24 h. The resulting mixture was cooled to ambient temperature, diluted with 15.0 mL of CH_2Cl_2, and washed with 10% NaOH (aq.,) until the aqueous phase became colorless. Then the organic layer was dried over Na_2SO_4 and concentrated under reduced pressure. Purification was conducted by column chromatography on silica gel (CH_2Cl_2/petroleum ether = 1:10, v/v) to give **1** as a yellow solid (11.3 mg, 21%). ^{1}H NMR (400 MHz, $CDCl_3$): δ (ppm) 9.25 (s, 4H), 8.99 (d, J = 8.8 Hz, 4H), 8.74 (d, J = 9.2 Hz, 4H), 7.38 (s, 4H), 2.66 (s, 6H), 1.89 (s, 12H). ^{13}C NMR (100 MHz, $CDCl_3$): δ (ppm) 138.32, 137.66, 135.68, 134.07, 128.67, 127.90, 127.28, 125.43, 125.39, 122.25, 120.64, 21.50, 20.44. The spectral data of **1** matched with previously reported literature[4].

2. Synthesis of 5,12-dimesityldibenzo[*bc*,*ef*]coronene (3)

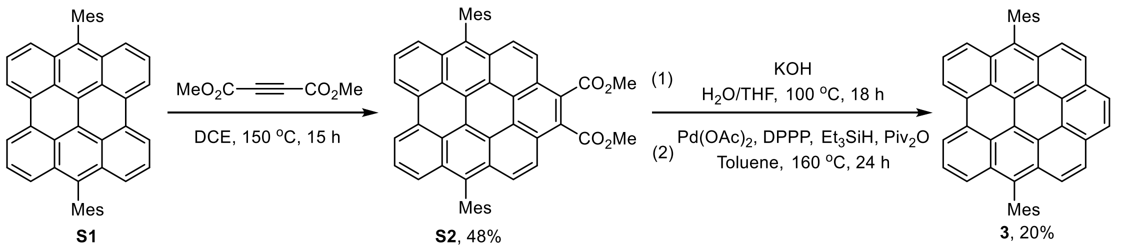

Step 1: A 25 mL Schlenk tube with a magnetic stir bar was charged with dimesitylphenanthro[1,10,9,8-*opqra*]perylene **S1** (28.4 mg, 0.05 mmol), dimethyl but-

2-ynedioate (7.2 μL, 0.05 mmol), and 1,2-dichloroethane (DCE) (0.5 mL) under a nitrogen atmosphere. The vessel was sealed tightly with a screw cap, then stirred at 150 °C in an oil bath for 15 h. After that, the resulting solution was cooled to ambient temperature, and DCE was evaporated under reduced pressure. Purification was conducted by column chromatography on silica gel (CH_2Cl_2/petroleum ether = 4:1, v/v) to give **S2** as a purplish red solid (16.7 mg, 48%). ^{1}H NMR (400 MHz, $CDCl_3$): δ (ppm) 9.17 (d, J = 7.2Hz, 2H), 8.33 (d, J = 9.6Hz, 2H), 8.04-7.95 (m, 4H), 7.78 (d, J = 9.6Hz, 2H), 7.21 (s, 4H), 4.12 (s, 6H), 2.53 (s, 6H), 1.89 (s, 12H). ^{13}C NMR (100 MHz, $CDCl_3$) δ (ppm) 169.30, 137.87, 137.80, 136.23, 134.50, 132.00, 131.16, 128.74, 128.66, 128.09, 127.29, 127.14, 126.78, 125.86, 125.60, 125.23, 124.76, 124.28, 122.27, 121.38, 53.59, 53.17, 21.49, 20.28. HRMS (ESI$^+$): calcd for $[C_{52}H_{38}O_4Na]^+$, 749.2662; found 749.2664.

Step 2: A 250 mL Schlenk tube with a magnetic stir bar was charged with **S2** (43.9 mg, 0.06 mmol) and KOH (28.0 mg, 0.5 mmol) in THF/H_2O (2.5 mL, 1:1, 4/1) under a nitrogen atmosphere. The vessel was sealed tightly with a screw cap, then stirred at 100 °C in an oil bath for 24 h. The resulting solution was cooled to ambient temperature, and THF was evaporated under reduced pressure. Then HCl (aq., 2.0 M) was added dropwise to the resulting mixture until the pH value was in a range of 1-2. The resulting mixture was stirred at 25 °C for 1 h. The precipitate was filtered, washed with THF/water (20 mL, 1:50, v/v), and dried to give the hydrolysis product, which was used directly in the next step.

Step 3: A 25 mL Schlenk tube with a magnetic stir bar was charged with $Pd(OAc)_2$ (5.2 mg, 0.023 mmol), 1,3-bis(diphenylphosphino)propane (DPPP) (16.8 mg, 0.04 mmol), Et_3SiH (16.0 μL, 0.1 mmol), Piv_2O (121.1 μL, 0.6 mmol), and hydrolysis product (69.9 mg, 0.1 mmol) in toluene (1.0 mL) under a nitrogen atmosphere. The vessel was sealed tightly with a screw cap, then stirred at 160 °C in an oil bath for 24 h. The resulting solution was cooled to ambient temperature, diluted with 10.0 mL of CH_2Cl_2, filtered through a celite pad, and washed three times with 10.0 mL of CH_2Cl_2. The obtained organic extracts were evaporated under reduced pressure. Purification was performed

by column chromatography on silica gel (petroleum ether) to provide **3** as an orange solid (12.2 mg, 20%). ^{1}H NMR (400 MHz, $CDCl_3$): δ (ppm) 9.11 (d, J = 7.2 Hz, 2H), 8.47 (s, 2H), 8.04 (d, J = 9.2 Hz, 2H), 7.98-7.91 (m, 4H), 7.67 (d, J = 9.2 Hz, 2H), 7.22 (s, 4H), 2.55 (s, 6H), 1.91 (s, 12H). ^{13}C NMR (100 MHz, $CDCl_3$) δ (ppm) 137.89, 137.41, 134.93, 134.66, 131.95, 130.73, 129.95, 129.04, 128.81, 128.55, 126.56, 125.89, 125.32, 125.08, 125.06, 123.64, 123.57, 122.65, 120.49, 21.38, 20.15. HRMS (ESI^+): calcd for $[C_{48}H_{35}]^+$, 611.2733; found 611.2738.

3. Synthesis of 7,18-dimesityldibenzo[*hi*,*kl*]naphtho[2,3-*a*]coronene (4)

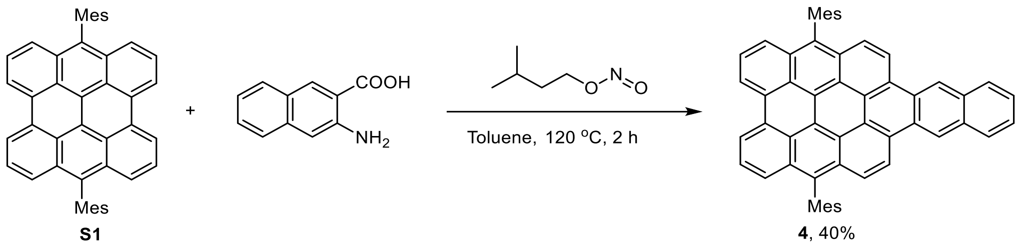

A 100 mL Schlenk tube with a magnetic stir bar was charged with 7,14-dimesitylphenanthro[1,10,9,8-*opqra*]perylene (**S1**) (28.4 mg, 0.048 mmol), 3-amino-2-naphthoic acid (9.4 mg, 0.05 mmol), and toluene (10.0 mL). Subsequently, isopentyl nitrite (20.0 μL, 0.15 mmol) was added dropwise to the mixture at ambient temperature. The vessel was sealed tightly with a screw cap, then stirred at 120 °C in an oil bath for 2 h. The resulting solution was cooled to ambient temperature, diluted with 10.0 mL of toluene, filtered through a celite pad, and washed three times with 10.0 mL of toluene. The obtained organic extracts were evaporated under reduced pressure. Purification was performed by column chromatography on silica gel (toluene/petroleum ether = 1:10, v/v) to provide **4** as an orange solid (13.6 mg, 40%). ^{1}H NMR (400 MHz, $CDCl_3$): δ (ppm) 9.65 (s, 2H), 9.11-8.87 (m, 4H), 8.28 (m, 2H), 7.79 (m, 4H), 7.63 (m, 4H), 7.24 (s, 4H), 2.56 (s, 6H), 1.96 (s, 12H). The compound exhibited poor stability, leading to degradation during the spectral collection process and resulting in the appearance of impurity peaks. ^{13}C NMR (100 MHz, $CDCl_3$) δ (ppm) 137.47, 128.64, 128.25, 125.74, 21.40, 20.18. The poor solubility of the compound prevented the effective acquisition

of its ^{13}C NMR spectrum. HRMS (ESI$^+$): calcd for $[C_{56}H_{38}]^+$, 710.2974; found 710.2978.

4. Construction of the red emitter library

Table S1. Scope of red emitters

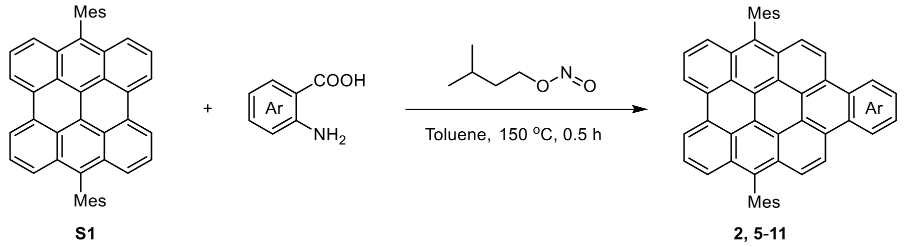

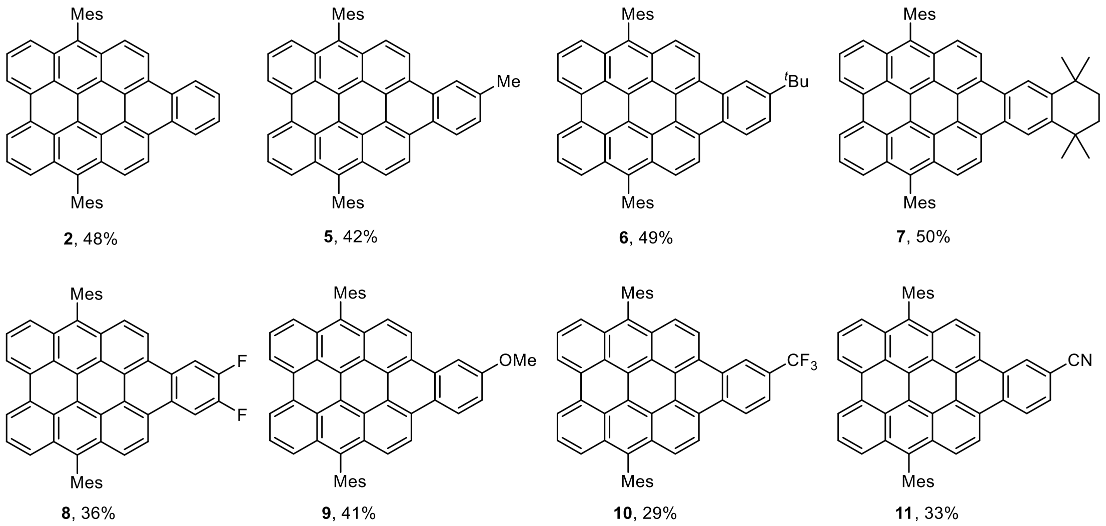

General procedure: A 100 mL Schlenk tube with a magnetic stir bar was charged with 7,14-dimesitylphenanthro[1,10,9,8-*opqra*]perylene (**S1**) (28.4 mg, 0.048 mmol), derivative of *o*-aminobenzoic acid (0.05 mmol), and toluene (10.0 mL). Subsequently, isopentyl nitrite (20.0 μL, 0.15 mmol) was added dropwise to the mixture at ambient temperature. The vessel was sealed tightly with a screw cap, then stirred at 150 °C in an oil bath for 0.5 h. The resulting solution was cooled to ambient temperature, diluted with 10.0 mL of CH_2Cl_2, filtered through a celite pad, and washed three times with 10.0 mL of CH_2Cl_2. The obtained organic extracts were evaporated under reduced pressure. Purification was performed by column chromatography on silica gel to provide desired products.

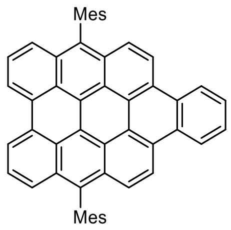

7,16-Dimesityltribenzo[*a*,*hi*,*kl*]coronene (**2**)

Following the general procedure, *o*-aminobenzoic acid was used (6.9 mg, 0.05 mmol). Purification was performed by column chromatography on silica gel (CH_2Cl_2/petroleum ether = 1:25, v/v) to provide **2** as a purple solid (15.2 mg, 48%). ^{1}H NMR (800 MHz, $CDCl_3$): δ (ppm) 9.19-9.18 (m, 2H), 8.99-8.98 (m, 4H), 7.97-7.96 (m, 2H), 7.86-7.85 (m, 6H), 7.24 (s, 4H), 2.56 (s, 6H), 1.94 (s, 12H). ^{13}C NMR (200 MHz, $CDCl_3$): δ (ppm) 138.05, 137.60, 135.10, 132.41, 128.75, 127.85, 126.38, 125.62, 124.66, 123.81, 123.65, 123.42, 120.61, 21.52, 20.28. HRMS (ESI$^+$): calcd for $[C_{52}H_{36}]^+$, 660.2817; found 660.2813.

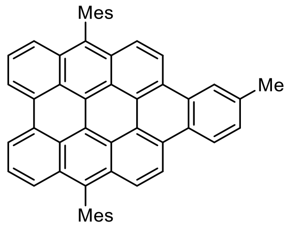

7,16-Dimesityl-11-methyltribenzo[*a*,*hi*,*kl*]coronene (**5**)

Following the general procedure, 2-amino-4-methylbenzoic acid was used (7.6 mg, 0.05 mmol). Purification was performed by column chromatography on silica gel (CH_2Cl_2/petroleum ether = 1:25, v/v) to provide **5** as a purple solid (13.6 mg, 42%). ^{1}H NMR (800 MHz, $CDCl_3$): δ (ppm) 9.05 (d, J = 8.0 Hz, 1H), 8.96-8.93 (m, 4H), 8.92 (s, 1H), 7.86-7.84 (m, 4H), 7.82-7.80 (m, 2H), 7.77 (d, J = 8.0 Hz, 1H), 7.24-7.23 (m, 4H), 2.79 (s, 3H), 2.56-2.55 (m, 6H), 1.94-1.93 (m, 12H). ^{13}C NMR (200 MHz, $CDCl_3$): δ (ppm) 137.94, 137.93, 137.42, 137.40, 136.01, 135.01, 134.99, 134.84, 134.68, 132.28, 132.25, 130.81, 130.73, 129.32, 129.19, 128.59, 128.39, 127.90, 126.67, 126.63, 126.31, 126.10, 125.93, 125.87, 125.80, 125.46, 125.41, 125.36, 124.51, 124.34, 123.74, 123.70, 123.43, 123.36, 122.71, 120.43, 120.37, 29.71, 22.39, 21.38, 20.14. HRMS (ESI$^+$): calcd for $[C_{53}H_{38}]^+$, 674.2974; found 674.2979.

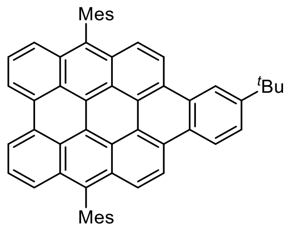

11-(*tert*-Butyl)-7,16-dimesityltribenzo[*a*,*hi*,*kl*]coronene (**6**)

Following the general procedure, 2-amino-4-(*tert*-butyl)benzoic acid was used (9.7 mg, 0.05 mmol). Purification was performed by column chromatography on silica gel (CH_2Cl_2/petroleum ether = 1:25, v/v) to provide **6** as a purple solid (16.9 mg, 49%). ^{1}H NMR (800 MHz, $CDCl_3$): δ (ppm) 9.16 (s, 1H), 9.13 (d, J = 8.8 Hz, 1H), 9.05 (d, J = 10.4 Hz, 1H), 8.97-8.95 (m, 3H), 8.07 (dd, J_1 = 8.8 Hz, J_2 = 1.6 Hz, 1H), 7.88-7.81 (m, 6H), 7.23-7.22 (m, 4H), 2.551-2.546 (m, 6H), 1.93 (m, 12H), 1.61 (s, 9H). ^{13}C NMR (200 MHz, $CDCl_3$): δ (ppm) 149.02, 138.07, 137.55, 137.54, 135.14, 134.96, 132.43, 132.40, 130.88, 129.37, 128.72, 128.71, 127.69, 126.78, 126.45, 126.32, 126.23, 126.12, 126.01, 125.60, 125.53, 125.27, 124.62, 124.50, 123.90, 123.85, 123.59, 123.49, 122.98, 120.55, 118.67, 118.64, 35.62, 31.67, 21.51, 21.50, 20.29, 20.26. HRMS (ESI$^+$): calcd for $[C_{56}H_{44}]^+$, 716.3443; found 716.3446.

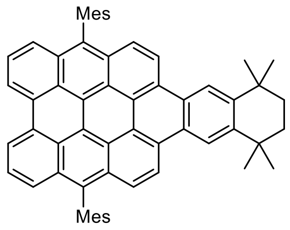

7,18-Dimesityl-11,11,14,14-tetramethyl-11,12,13,14

tetrahydrodibenzo[*hi*,*kl*]naphtho[2,3-*a*]coronene (**7**)

Following the general procedure, 3-amino-5,5,8,8-tetramethyl-5,6,7,8-tetrahydronaphthalene-2-carboxylic acid was used (12.4 mg, 0.05 mmol). Purification was performed by column chromatography on silica gel (CH_2Cl_2/petroleum ether = 1:25, v/v) to provide **7** as a purple solid (18.5 mg, 50%). ^{1}H NMR (800 MHz, $CDCl_3$): δ (ppm) 9.12 (s, 1H), 8.98 (d, J = 8.8 Hz, 2H), 8.924-8.918 (m, 2H), 7.84-7.80 (m, 6H), 7.21 (s, 4H), 2.54 (s, 6H), 1.93 (s, 4H), 1.92 (s, 12H), 1.58 (s, 12H). ^{13}C NMR (200 MHz, $CDCl_3$): δ (ppm) 144.71, 138.04, 137.49, 135.16, 134.83, 132.43, 130.88, 129.45,

128.69, 126.72, 126.27, 125.99, 125.63, 125.50, 124.56, 124.01, 123.53, 123.08, 120.75, 120.46, 35.44, 35.29, 33.05, 21.49, 20.26. HRMS (ESI$^+$): calcd for $[C_{60}H_{50}]^+$, 770.3913; found 770.3916.

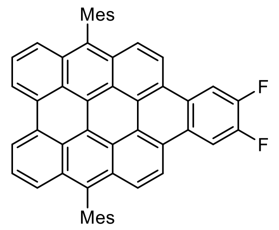

11,12-Difluoro-7,16-dimesityltribenzo[*a*,*hi*,*kl*]coronene (**8**)

Following the general procedure, 2-amino-4,5-difluorobenzoic acid was used (14.5 mg, 0.05 mmol). Purification was performed by column chromatography on silica gel (CH_2Cl_2/petroleum ether = 1:25, v/v) to provide **8** as a purple solid (12.0 mg, 36%). ^{1}H NMR (800 MHz, $CDCl_3$): δ (ppm) 8.98 (d, J = 7.2 Hz, 2H), 8.85 (t, J = 9.6 Hz, 2H), 8.71 (d, J = 9.6 Hz, 2H), 7.86-7.87 (m, 4H), 7.84-7.83 (m, 2H), 7.24 (s, 4H), 2.55 (s, 6H), 1.92 (s, 12H). ^{13}C NMR (200 MHz, $CDCl_3$): δ (ppm) 150.92, 150.84, 149.78, 149.66, 149.58, 137.99, 137.74, 135.48, 134.84, 132.37, 131.08, 129.33, 128.80, 127.06, 126.87, 125.70, 125.66, 125.10, 124.65, 123.55, 123.49, 123.28, 120.90, 110.50, 110.44, 21.52, 20.27. HRMS (ESI$^+$): calcd for $[C_{52}H_{34}F_2]^+$, 696.2629; found 696.2631.

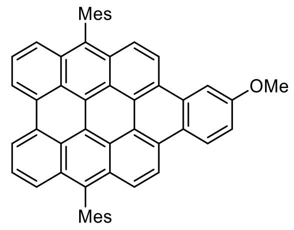

7,16-Dimesityl-11-methoxytribenzo[*a*,*hi*,*kl*]coronene (**9**)

Following the general procedure, 2-amino-4-methoxybenzoic acid was used (8.4 mg, 0.05 mmol). Purification was performed by column chromatography on silica gel (CH_2Cl_2/petroleum ether = 1:15, v/v) to provide **9** as a purple solid (13.6 mg, 41%). ^{1}H NMR (800 MHz, $CDCl_3$): δ (ppm) 9.08 (d, J = 10.4 Hz, 1H), 8.96 (t, J = 6.4 Hz, 2H), 8.90 (d, J = 9.6 Hz, 1H), 8.86 (d, J = 8.8 Hz, 1H), 8.42 (d, J = 2.4 Hz, 1H), 7.86-7.81 (m, 6H), 7.61 (dd, J_1 = 8.8 Hz, J_1 = 2.4 Hz, 1H), 7.23 (m, 4H), 4.12 (s, 3H), 2.55 (m, 6H), 1.94-1.93 (m, 12H). ^{13}C NMR (200 MHz, $CDCl_3$): δ (ppm) 158.29, 138.06, 137.57,

137.54, 135.13, 135.11, 134.66, 132.46, 132.34, 131.02, 130.76, 129.55, 129.44, 129.09, 128.73, 126.85, 126.71, 126.63, 126.46, 125.92, 125.61, 125.60, 125.43, 125.38, 124.77, 124.21, 123.89, 123.83, 123.81, 123.54, 123.48, 123.05, 121.99, 120.64, 120.46, 118.21, 102.90, 102.88, 55.62, 21.51, 20.28, 20.27. HRMS (ESI$^+$): calcd for $[C_{53}H_{38}O]^+$, 690.2923; found 690.2917.

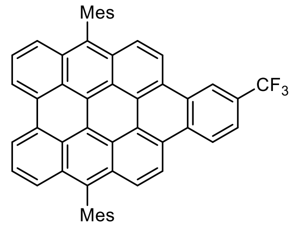

7,16-Dimesityl-11-(trifluoromethyl)tribenzo[*a*,*hi*,*kl*]coronene (**10**)

Following the general procedure, 2-amino-4-(trifluoromethyl)benzoic acid was used (10.3 mg, 0.05 mmol). Purification was performed by column chromatography on silica gel (CH_2Cl_2/petroleum ether = 1:20, v/v) to provide **10** as a purple solid (10.1 mg, 29%). ^{1}H NMR (800 MHz, $CDCl_3$): δ (ppm) 9.48 (s, 1H), 9.29 (d, J = 8.8 Hz, 1H), 9.00-8.96 (m, 4H), 8.13 (d, J = 8.8 Hz, 1H), 7.94 (d, J = 9.6 Hz, 1H), 7.92 (d, J = 8.8 Hz, 1H), 7.90-7.88 (m, 1H), 7.86-7.84 (m, 1H), 7.24 (m, 4H), 2.56 (m, 6H), 1.930-1.925 (m, 12H). ^{13}C NMR (200 MHz, $CDCl_3$): δ (ppm) 138.00, 137.96, 137.77, 137.75, 135.79, 135.54, 134.84, 134.77, 132.41, 132.37, 131.21, 131.13, 129.55, 129.44, 129.06, 128.80, 127.80, 127.64, 127.18, 127.14, 126.94, 126.71, 126.67, 125.97, 125.82, 125.73, 125.67, 124.94, 124.84, 124.80, 124.74, 124.56, 124.31, 124.02, 123.54, 123.50, 123.37, 123.12, 121.76, 121.45, 121.03, 120.95, 21.51, 20.28, 20.27. HRMS (ESI$^+$): calcd for $[C_{53}H_{35}F_3]^+$, 728.2691; found 728.2693.

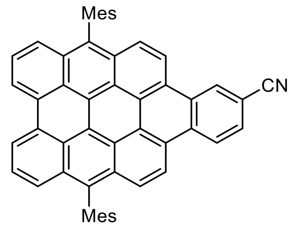

7,16-Dimesityltribenzo[*a*,*hi*,*kl*]coronene-11-carbonitrile (**11**)

Following the general procedure, 2-amino-4-cyanobenzoic acid was used (8.1 mg, 0.05 mmol). Purification was performed by column chromatography on silica gel

(CH_2Cl_2/petroleum ether = 1:15, v/v) to provide **11** as a purple solid (10.9 mg, 33%). ^{1}H NMR (800 MHz, $CDCl_3$): δ (ppm) 9.49 (s, 1H), 9.19 (d, J = 8.8 Hz, 1H), 9.02-9.00 (m, 2H), 8.89-8.86 (m, 4H), 8.01 (dd, J_1 = 8.8 Hz, J_2 = 0.8 Hz, 1H), 7.95 (d, J = 9.6 Hz, 1H), 7.92-7.89 (m, 3H), 7.88-7.85 (m, 2H), 7.25-7.24 (m, 4H), 2.57-2.56 (m, 6H), 1.93 (m, 12H). ^{13}C NMR (200 MHz, $CDCl_3$): δ (ppm) 137.96, 137.93, 137.88, 137.83, 136.15, 135.76, 134.71, 134.64, 132.40, 132.33, 131.34, 131.20, 129.89, 129.56, 129.40, 129.02, 128.87, 128.84, 127.55, 127.35, 127.28, 127.18, 126.59, 126.47, 126.14, 125.94, 125.80, 125.71, 125.69, 125.07, 125.04, 124.89, 124.85, 124.76, 124.11, 123.40, 123.34, 123.16, 122.78, 121.26, 121.13, 119.97, 109.22, 21.52, 20.30, 20.28. HRMS (ESI$^+$): calcd for $[C_{53}H_{35}N]^+$, 685.2770; found 685.2770.

IV. Photophysical Properties

Table S2. Summary of photophysical properties of red-emitting molecules

Emitter	$\lambda_{abs}{}^a$ [nm]	ε^a [L M^{-1} cm^{-1}]	$\lambda_{em}{}^a$ [nm]	FWHM^a [nm]/ [eV]	$\Phi_{PL}{}^a$ [%]	CIE^a [x, y]	τ^a [ns]	$k_r{}^a$ [s^{-1}]
**2**	615	6.5×10^4	621, 671 (0.23^b)	21/ 0.067	71	0.696, 0.304	8.6	8.3×10^7
**5**	616	6.5×10^4	622, 672 (0.23^b)	21/ 0.067	74	0.697, 0.303	8.6	8.6×10^7
**6**	614	5.9×10^4	622, 672 (0.22^b)	22/ 0.070	73	0.696, 0.304	8.5	8.6×10^7
**7**	622	7.5×10^4	630, 680 (0.23^b)	21/ 0.066	76	0.707, 0.293	8.9	8.5×10^7
**8**	612	5.9×10^4	618, 667 (0.23^b)	22/ 0.071	82	0.691, 0.309	8.6	9.5×10^7
**9**	615	6.0×10^4	626, 675 (0.21^b)	24/ 0.076	62	0.701, 0.299	8.5	7.3×10^7
**10**	616	6.1×10^4	624, 674 (0.22^b)	22/ 0.076	65	0.700, 0.300	9.0	7.2×10^7
**11**	622	5.7×10^4	630, 681 (0.20^b)	23/ 0.072	69	0.707, 0.293	9.1	7.6×10^7

^{a}Measured in toluene solution (1.0×10^{-5} M). ^{b}Relative intensity of the shoulder peak.

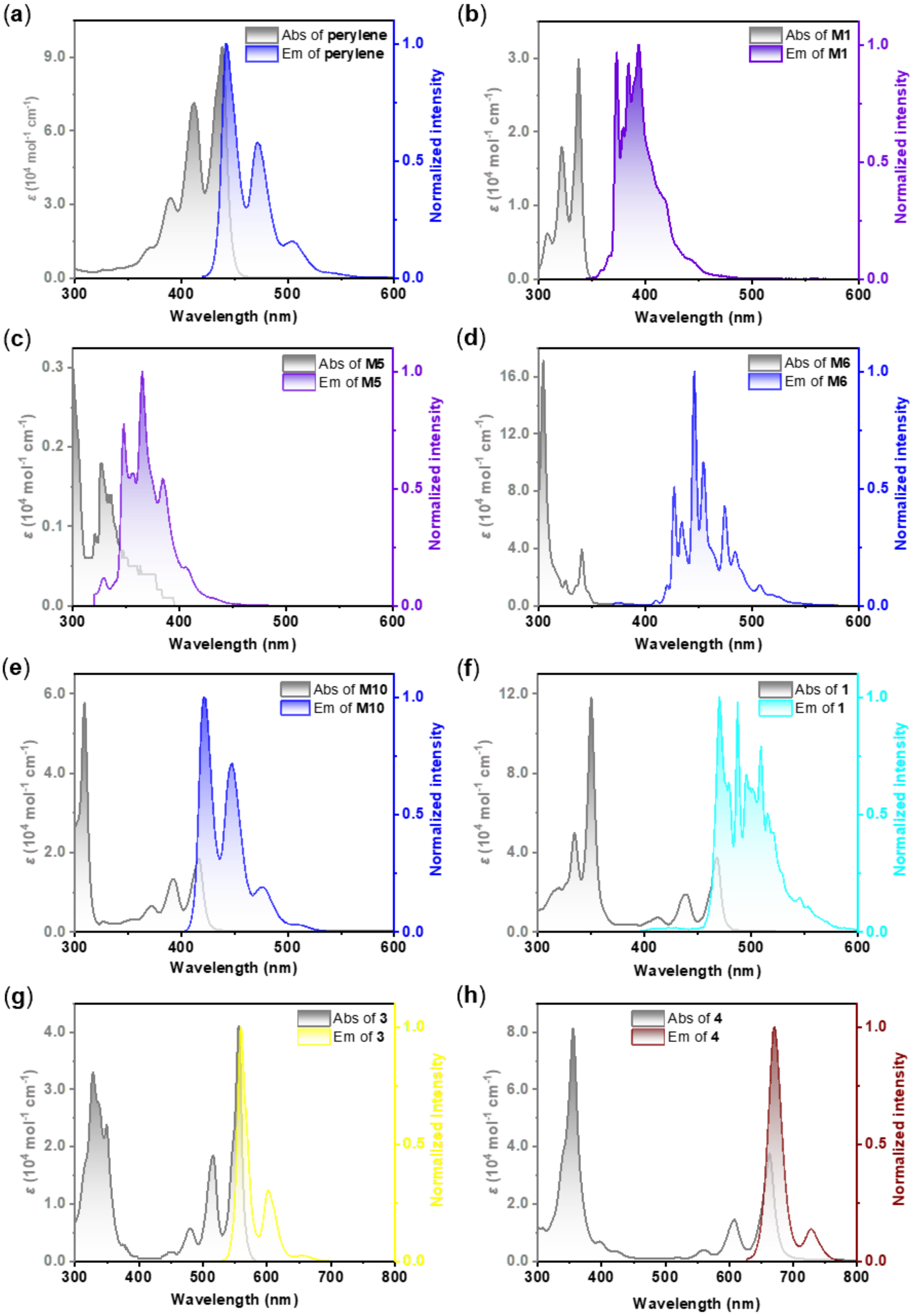

Figure S7. UV-vis absorption and fluorescence emission spectra of target molecules. (a) Compound **perylene**. (b) Compound **M1**. (c) Compound **M5**. (d) Compound **M6**. (e) Compound **M10**. (f) Compound **1**. (g) Compound **3**. (h) Compound **4**.

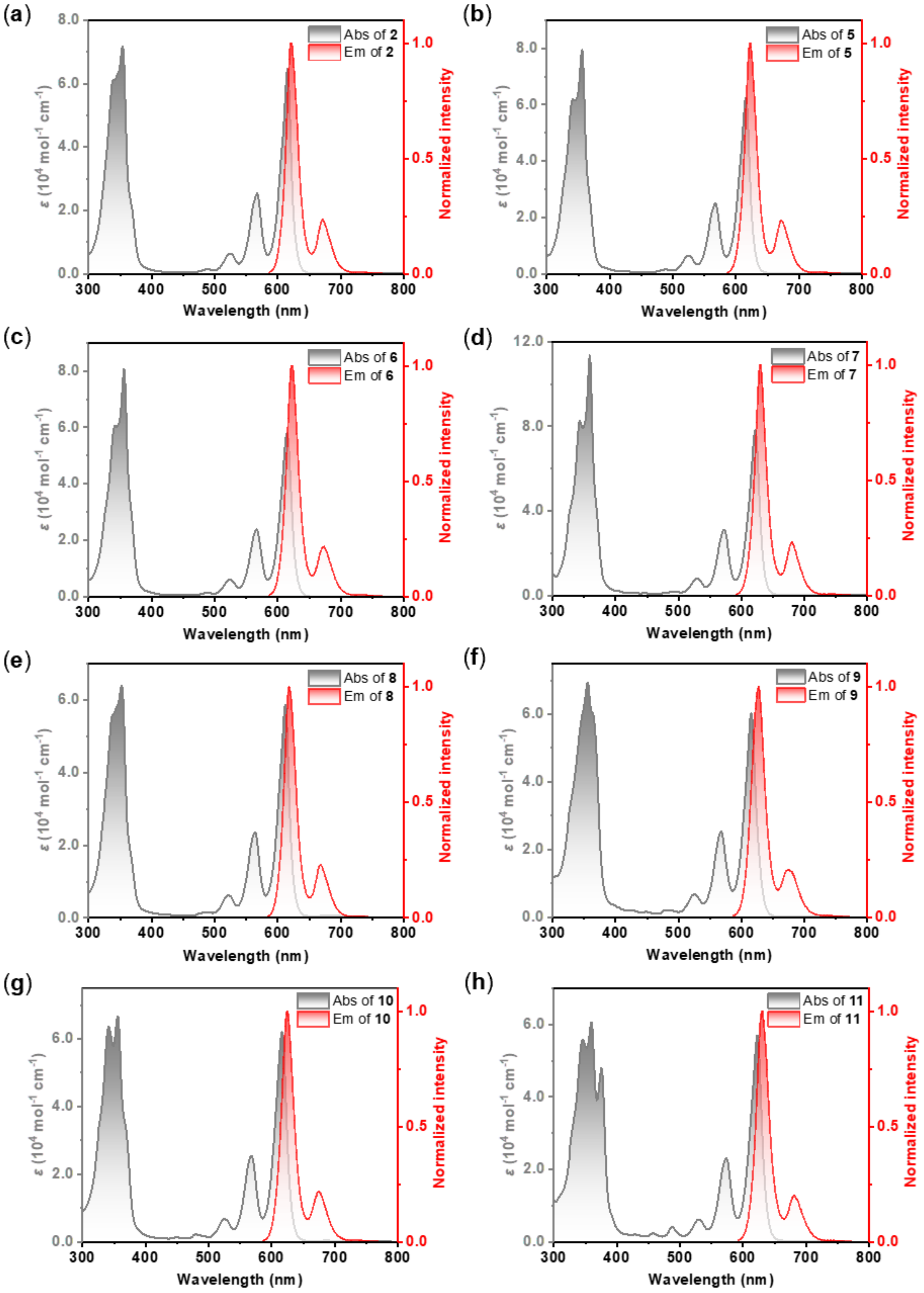

Figure S8. UV-vis absorption and emission spectra of red emitters. (a) Emitter **2**. (b) Emitter **5**. (c) Emitter **6**. (d) Emitter **7**. (e) Emitter **8**. (f) Emitter **9**. (g) Emitter **10**. (h) Emitter **11**.

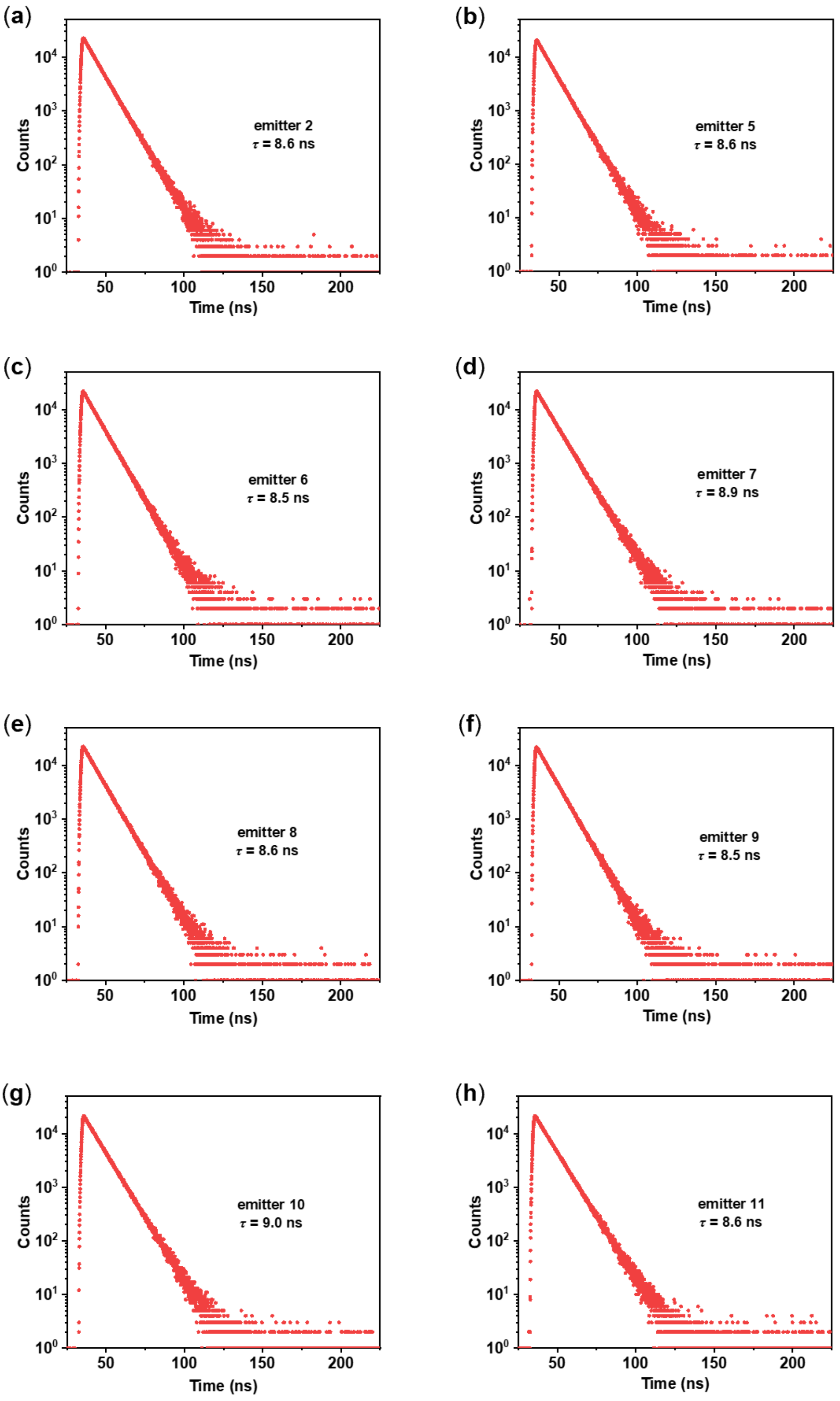

Figure S9. Transient photoluminescent decay spectra of red emitters. (a) Emitter **2**. (b) Emitter **5**. (c) Emitter **6**. (d) Emitter **7**. (e) Emitter **8**. (f) Emitter **9**. (g) Emitter **10**. (h) Emitter **11**.

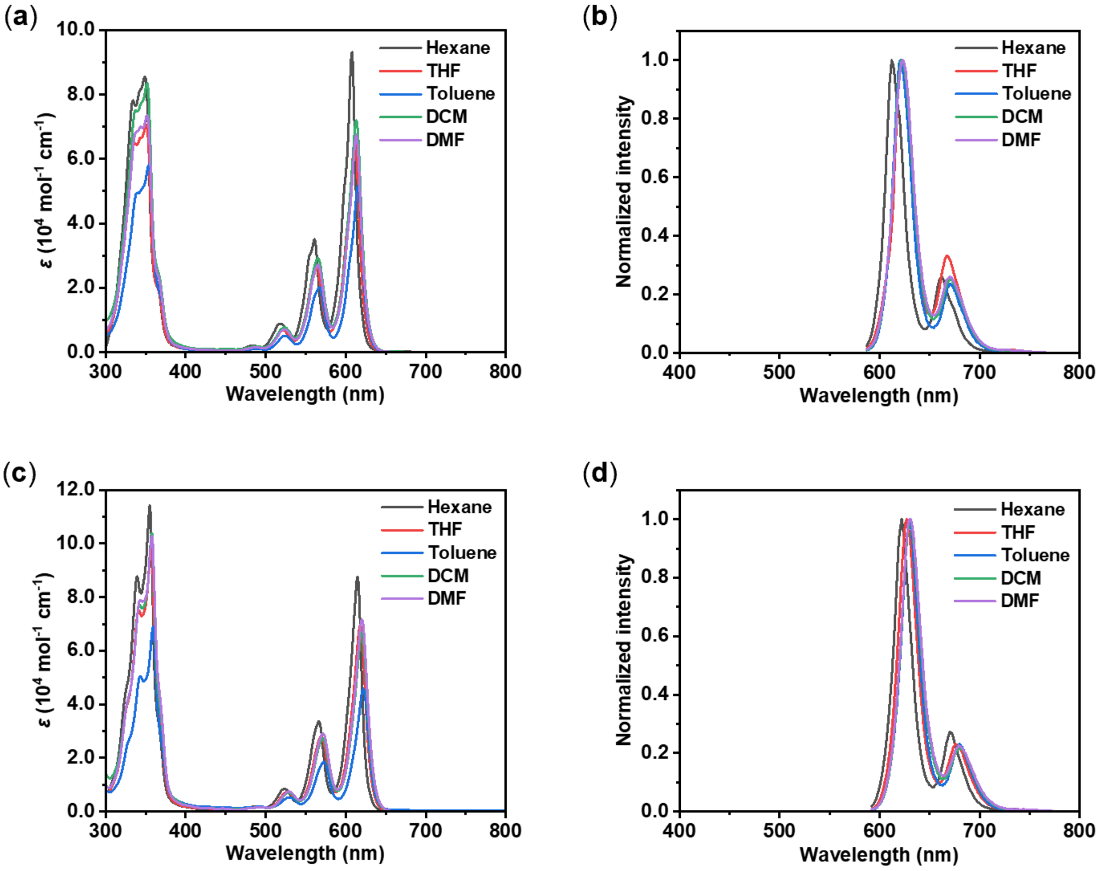

Figure S10. Absorption and fluorescence emission spectra of **2** and **7** in different solvents. (a) Absorption spectra of **2**. (b) Fluorescence emission spectra of **2**. (c) Absorption spectra of **7**. (d) Fluorescence emission spectra of **7**.

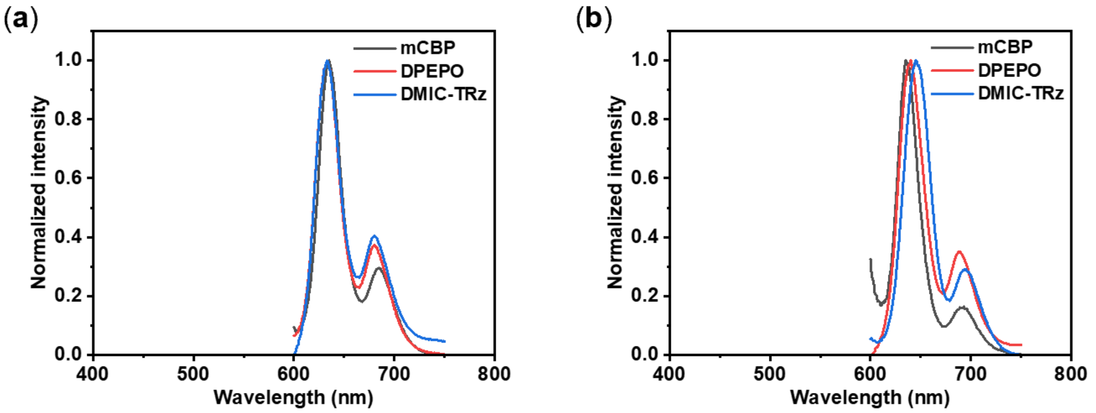

Figure S11. Fluorescence emission spectra of compounds **2** and **7** in thin films. (a) Compound **2**. (b) Compound **7**. All thin films were prepared by doping 1 wt% of the emitter into the host material.

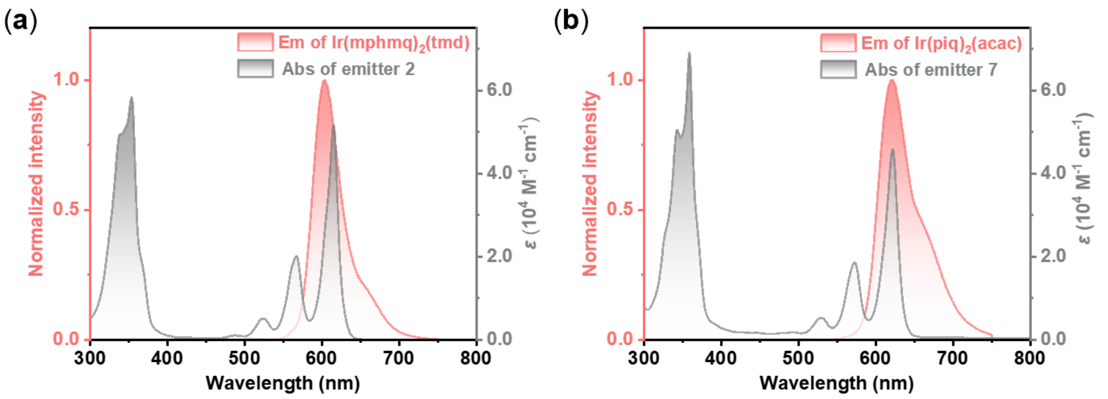

Figure S12. Comparison of the emission spectra of the phosphorescence sensitizers with the absorption and emission spectra of the emitters. (a) Ir(mphmq)2(tmd) and emitter **2**. (b) Ir(piq)2(acac) and emitter **7**.

V. Cyclic Voltammograms

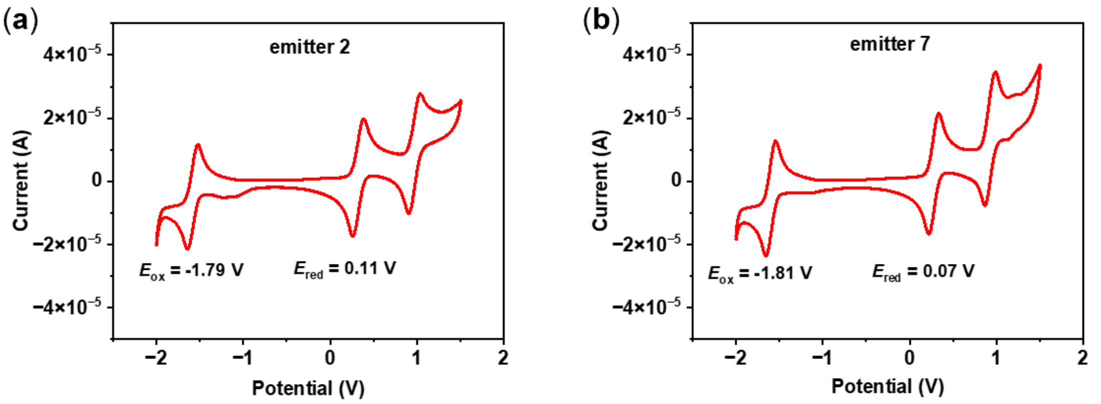

Figure S13. Cyclic voltammograms curves. (a) Emitter **2**. (b) Emitter **7**. The HOMO and LUMO energy levels were determined using $E_{HOMO} = -4.8-(E_{ox}-E_{Fc/Fc^+})$ and $E_{HOMO} = -4.8-(E_{red}-E_{Fc/Fc^+})$, respectively. The E_{Fc/Fc^+} was 0.21 V.

VI. Thermogravimetric Analysis

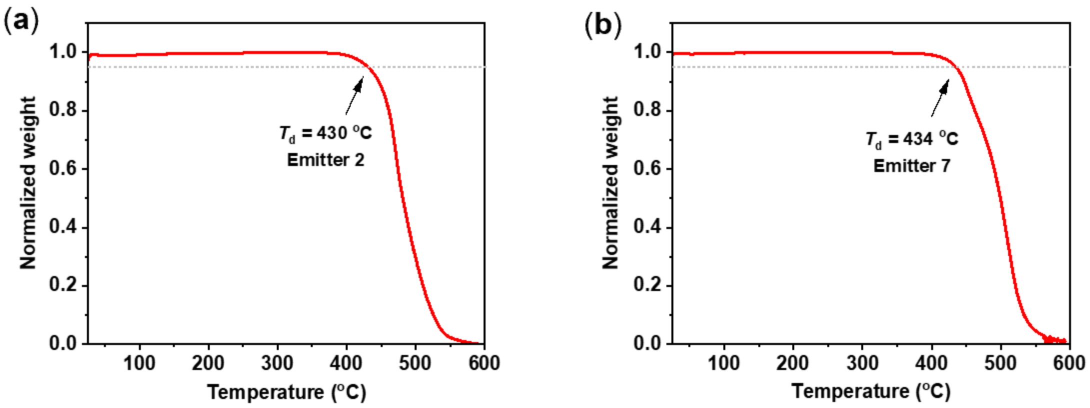

Figure S14. Thermogravimetric analysis curves. (a) Emitter **2**. (b) Emitter **7**.

VII. Characterization of OLED devices.

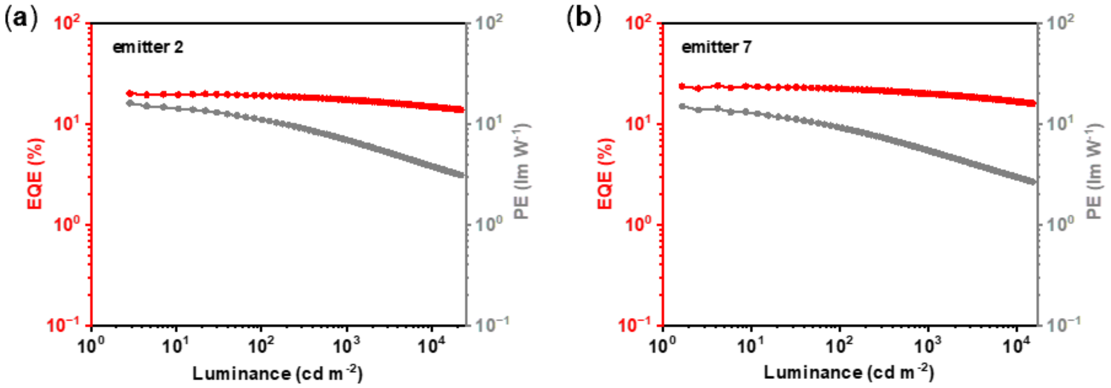

Figure S15. External quantum efficiency–luminance and current efficiency–luminance curves of optimized device. (a) Emitter **2**. (b) Emitter **7**.

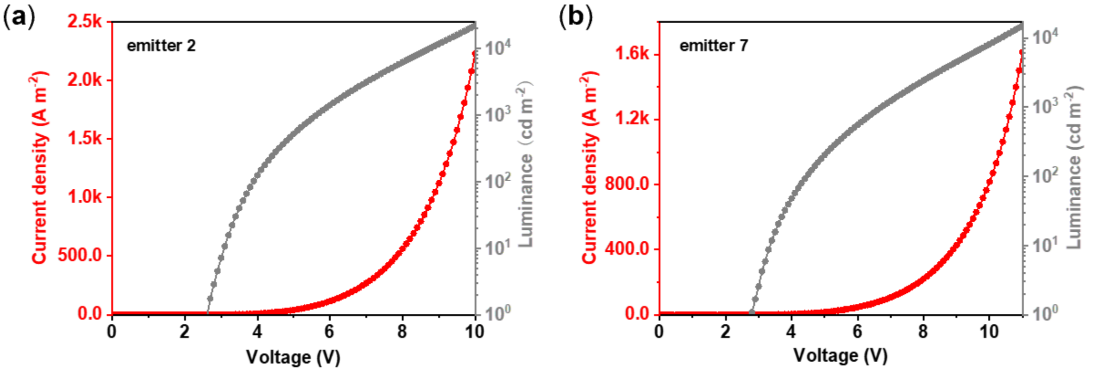

Figure S16. Current density and luminance versus voltage (*J-V-L*) characteristics of optimized device. (a) Emitter **2**. (b) Emitter **7**.

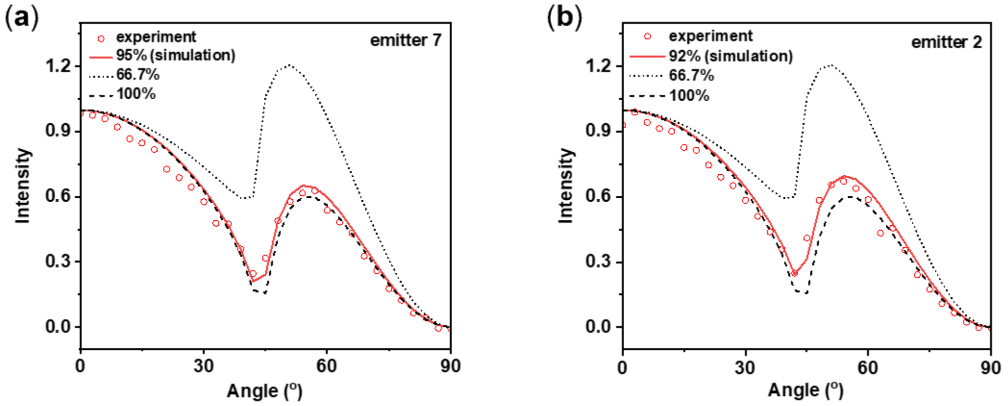

Figure S17. Angle-dependent PL intensity of the *p*-polarized light of the emitting layer. (a) 0.5% Emitter **2** doped in DMIC-TRz. (b) 0.8% Emitter **7** doped in DMIC-TRz.

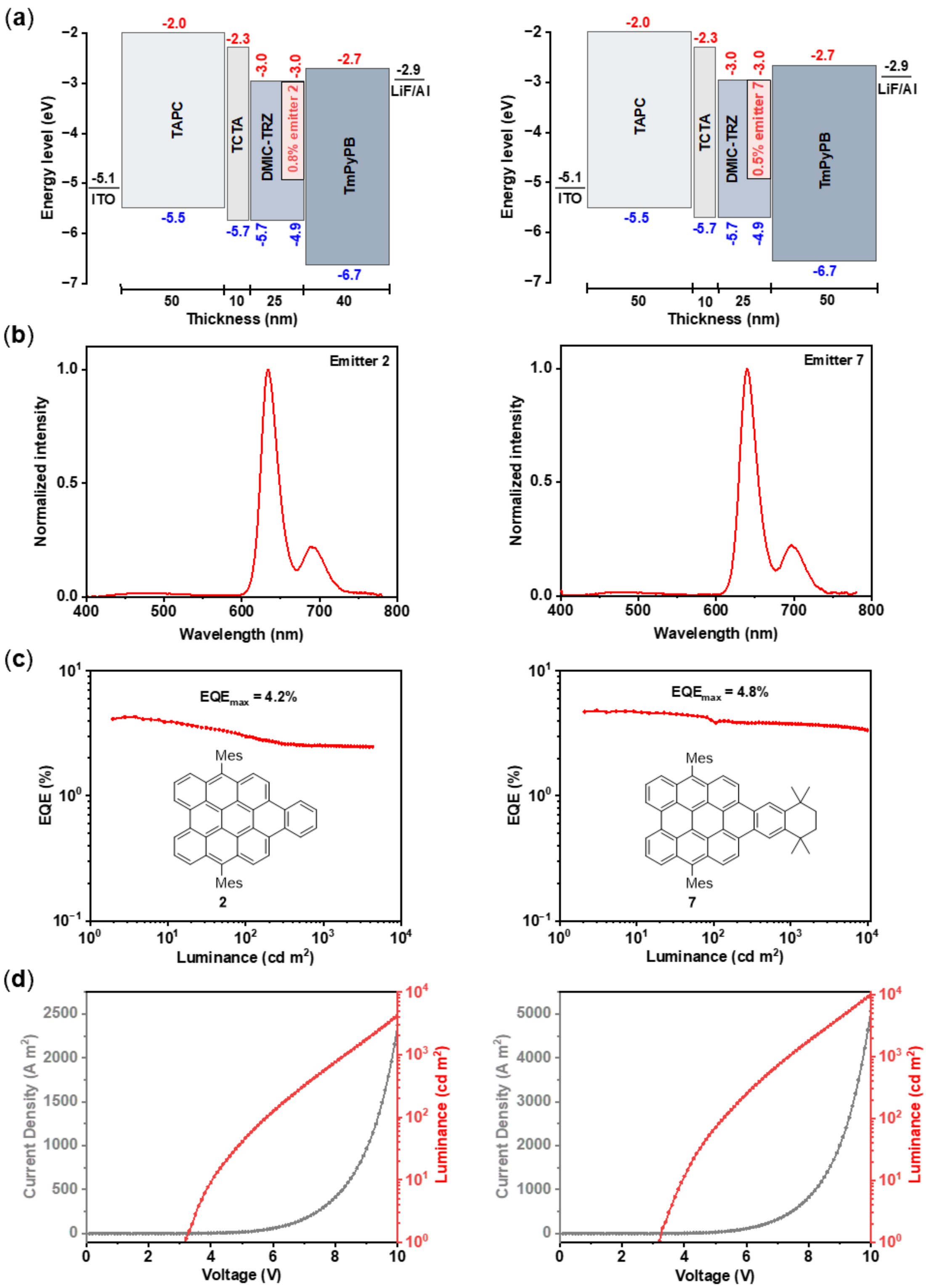

Figure S18. Research on OLED device based on the binary EML system. (a) Device architecture and energy-level diagram of the functional materials for OLEDs. (b) The electroluminescence spectra. (c) External quantum efficiency–luminance curve of device and chemical structure. (d) Current density and luminance versus voltage (*J*-*V*-*L*) characteristics of device. Left: emitter **2**; right: emitter **7**.

VIII. Performance Summary of the Reported Red Emitters

Table S3. Summary of the performance of traditional red emitters in OLEDs[a]

Emitter	CIE [x, y]	λ_{EL} [nm]	FWHM [nm]	EQE [%]	Reference
**BDP-AN**	0.67, 0.33	672	28	16.43	5
**B3**	0.67, 0.33	632	52	1.01	6
**α-EtNAICZ**	0.68, 0.32	633	56	11.5	7
**MeBF-PZ**	0.67, 0.33	623	30	18.3	8
***p*-OMB**	0.67, 0.33	644	53	1.3	9
**DPNA**	0.70, 0.30	666	40	12.23	10
**DPNA-F**	0.70, 0.30	661	40	12.08	10
**DPNA-tBu**	0.71, 0.29	667	40	11.40	10
**II-b**	0.71, 0.29	656	61	22.6	11
**7**	**0.704, 0.294**	**639**	**28**	**24.2**	**This work**

[a]Devices with a CIE (*x*) value ≥ 0.70 (NTSC standard) are considered.

Table S4. Summary of emitters approaching BT.2020 red standard in OLEDs[a]

Entry[b]	Emitter	CIE [x, y]	λ_{EL} [nm]	FWHM [nm]	EQE [%]	Reference
1	**DCPPr-TPA**	0.70, 0.29	734	105[c]	1.4	12
2	**TPA-DCPP**	0.70, 0.29	710	150[c]	2.1	13
3	**TPA-PPDCN**	0.70, 0.30	692	115[c]	16.4	14
4	***o*-QxDC-DTPA**	0.70, 0.30	680	160[c]	5.21	15
5	**T-*β*-IQD**	0.71, 0.29	711	125[c]	9.44	16
6	**Ir-G1**	0.70, 0.30	640	80[c]	11.65	17
7	**Ir-G2**	0.70, 0.30	640	80[c]	7.36	17
8	**$(DPQ)_2Ir(dpm)$**	0.70, 0.29	670	68[c]	17.9	18
9	**$(NAPQ)_2Ir(acac)$**	0.71, 0.29	642	35	2.1	19
10	**Ir1**	0.714, 0.285	660	60[c]	16.6	20
11	**Ir2**	0.715, 0.285	662	58[c]	14.6	20
12	**2S-BN**	0.70, 0.28	680	62	28.2	21
13	**CzIDBNO**	0.701, 0.298	643	47	32.5	22
14	**IDIDBNO**	0.702, 0.297	671	49	27.2	22
15	**BNNO**	0.708, 0.292	643	42	34.4	23
16	**RBNO2**	0.708, 0.292	648	49	26.6	24
17	**DPNA**	0.70, 0.30	666	40	12.23	10
18	**DPNA-F**	0.70, 0.30	661	40	12.08	10
19	**DPNA-^{t}Bu**	0.71, 0.29	667	40	11.40	10
20	**II-b**	0.71, 0.29	656	61	22.6	11
-	**7**	0.704, 0.294	639	28	24.2	This work

[a]Devices with a CIE (x) value ≥ 0.70 (BT.2020 standard) are considered. [b]The number in Figure 1b (iii). [c]Estimated values based on the graph (not provided by author).

Table S5. Chemical structure of red emitters mentioned in Table S2 and Table S3

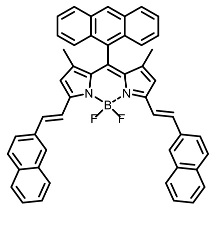

BOP-AN

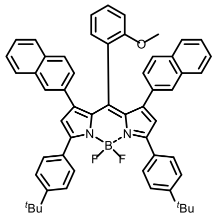

B3

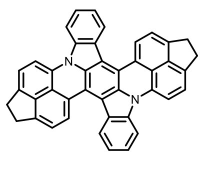

***α*-EtNAICZ**

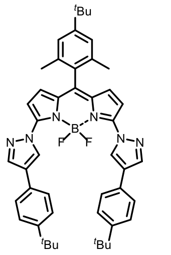

MeBF-PZ

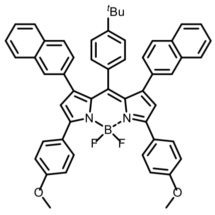

***p*-OMB**

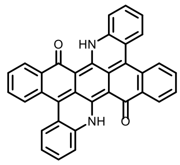

DPNA

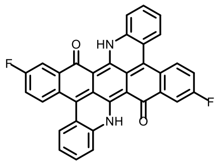

DPNA-F

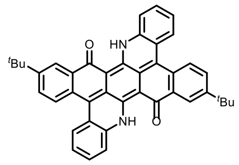

DPNA-^{t}Bu

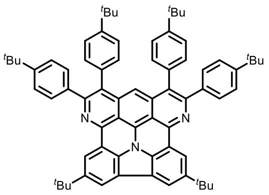

II-b

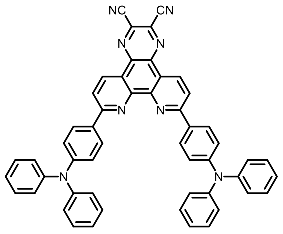

DCPPr-TPA

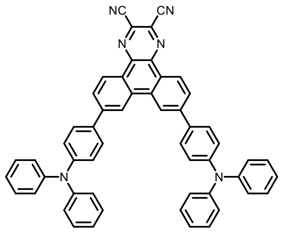

TPA-DCPP

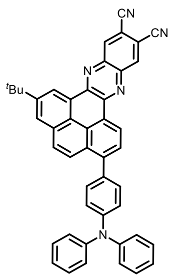

TPA-PPDCN

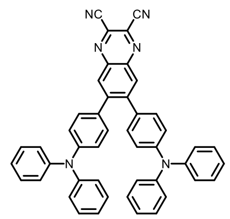

***o*-QxDC-DTPA**

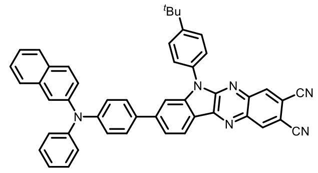

T-*β*-IQD

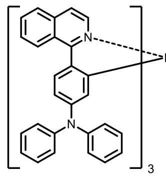

Ir-G1

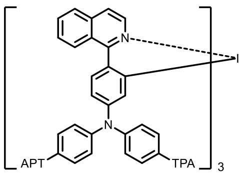

Ir-G2

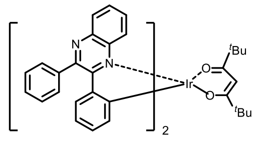

$(DPQ)_2Ir(dpm)$

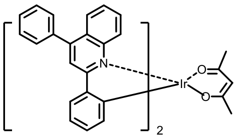

$(NAPQ)_2Ir(acac)$

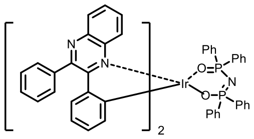

Ir1

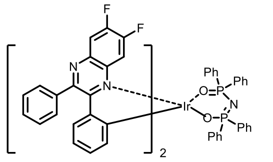

Ir2

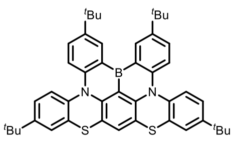

2S-BN

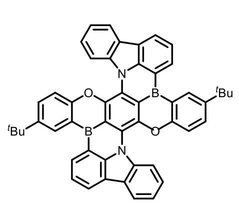

CzIDBNO

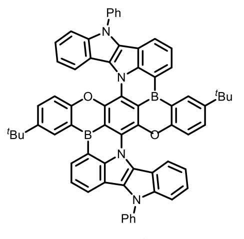

IDIDBNO

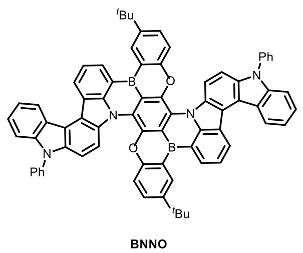

BNNO

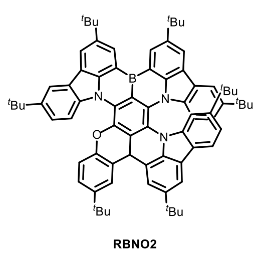

RBNO2

IX. Reference

1. Fort EH, Donovan PM, Scott LT. Diels−Alder Reactivity of polycyclic aromatic hydrocarbon bay regions: Implications for metal-free growth of single-chirality carbon nanotubes. *J Am Chem Soc* 2009; **131**: 16006−7.
2. Frisch MJ, Trucks GW, Schlegel HB *et al*. Gaussian 09, Revision D.01; Gaussian, Inc.: Wallingford, CT, 2009.
3. Lu T and Chen F. Multiwfn: A multifunctional wavefunction analyzer, *J Comput Chem* 2012; **3**: 580−92.
4. Fort EH and Scott LT. One-step conversion of aromatic hydrocarbon bay regions into unsubstituted benzene rings: A reagent for the low-temperature, metal-free growth of single-chirality carbon nanotubes. *Angew Chem Int Ed* 2010; **49**: 6626−8.
5. Nie X, Liu D, Li M *et al*. Boron-dipyrromethene emitters with strengthened rigidity and restricted scissoring vibration for high-performance OLEDs with deep-red narrowband emission. *Adv Optical Mater* 2025; **13**: 2402333.
6. Ma D, Zhao G, Chen H *et al*. Creation of BODIPYs-based red OLEDs with high color purity via modulating the energy gap and restricting rotation of substituents. *Dyes Pigm* 2022; **203**: 110377.
7. Zeng X, Wang X, Zhang Y *et al*. Nitrogen-embedded multi-resonance heteroaromatics with prolonged homogeneous hexatomic rings. *Angew Chem Int Ed* 2022; **61**: e202117181.
8. Liu J, Liu J, Li H *et al*. Boron-dipyrromethene-based fluorescent emitters enable high-performance narrowband red organic light-emitting diodes. *Angew Chem Int Ed* 2023; **62**: e202306471.
9. Jiang W, Zhou R, Zhao G *et al*. Isomer engineering to adjusting full width at half maximum and emission wavelength for efficient solution-processed red OLEDs. *Opt Mater* 2023; **136**: 113505.
10. Wu X, Wang C-H, Ni S *et al*. Multiple enol−keto isomerization and excited-state unidirectional intramolecular proton transfer generate intense, narrowband red OLEDs. *J Am Chem Soc* 2024; **146**: 24526−36.

11. Wu Y, Liu J, Yang G *et al*. Aromaticity localization effects in polycyclic aromatic hydrocarbons for discovering narrowband fluorescence materials. *J Am Chem Soc* 2025; **147**: 19305−14.
12. Cai Z, Wu X, Liu H *et al*. Realizing record-high electroluminescence efficiency of 31.5% for red thermally activated delayed fluorescence molecules. *Angew Chem Int Ed* 2021; **60**: 23635−40.
13. Wang S, Yan X, Cheng Z *et al*. Highly efficient near-infrared delayed fluorescence organic light emitting diodes using a phenanthrene-based charge-transfer compound. *Angew Chem Int Ed* 2015; **54**: 13068−72.
14. Yang T, Liang B, Cheng Z *et al*. Construction of efficient deep-red/near-infrared emitter based on a large π-conjugated acceptor and delayed fluorescence OLEDs with external quantum efficiency of over 20%. *J Phys Chem C* 2019; **123**: 18585−92.
15. Liu W, Liu Z, Yan J *et al*. A quinoxaline-based charge-transfer compound for efficient deep-red organic light emitting diodes. *Dyes Pigm* 2021; **191**: 109305.
16. Zhao M, Li M, Li W *et al*. Highly efficient near-infrared thermally activated delayed fluorescent emitters in non-doped electroluminescent devices. *Angew Chem Int Ed* 2022; **61**: e202210687.
17. Zhou G, Wong W-Y, Yao B *et al*. Triphenylamine-dendronized pure red iridium phosphors with superior OLED efficiency/color purity trade-offs. *Angew Chem Int Ed* 2007; **46**: 1149−51.
18. Nagai Y, Sasabe H, Takahashi J *et al*. Highly efficient, deep-red organic light-emitting devices using energy transfer from exciplexes. *J Mater Chem C* 2017; **5**: 527−30.
19. Ding J, Gao J, Fu Q *et al*. Highly efficient phosphorescent bis-cyclometalated iridium complexes based on quinoline ligands. *Synth Met* 2005; **155**: 539−48.
20. Jing Y-M, Wang F-Z, Zheng Y-X *et al*. Efficient deep red electroluminescence of iridium(III) complexes with 2,3-diphenylquinoxaline derivatives and tetraphenylimidodiphosphinate. *J Mater Chem C* 2017; **5**: 3714−24.

21. Pu Y, Jin Q, Zhang Y *et al*. Sulfur-locked multiple resonance emitters for high performance orange-red/deep red OLEDs. *Nat Commun* 2025; **16**: 332.

22. Jing Y-Y, Li N, Cao X et al. Precise modulation of multiple resonance emitters toward efficient electroluminescence with pure-red gamut for high-definition displays. *Sci Adv* 2023; **9**: eadh8296.

23. Fan T, Du M, Jia X *et al*. High-efficiency narrowband multi-resonance emitter fusing indolocarbazole donors for BT. 2020 red electroluminescence and ultralong operation lifetime. *Adv Mater* 2023; **35**: 2301018.

24. Zou Y, He J, Li N *et al*. Precisely regulating the double-boron-based multi-resonance framework towards pure-red emitters: High-performance OLEDs with CIE coordinates fully satisfying the BT. 2020 standard. *Mater Horiz* 2023; **10**: 3712−3718.

X. Copies of NMR Spectra

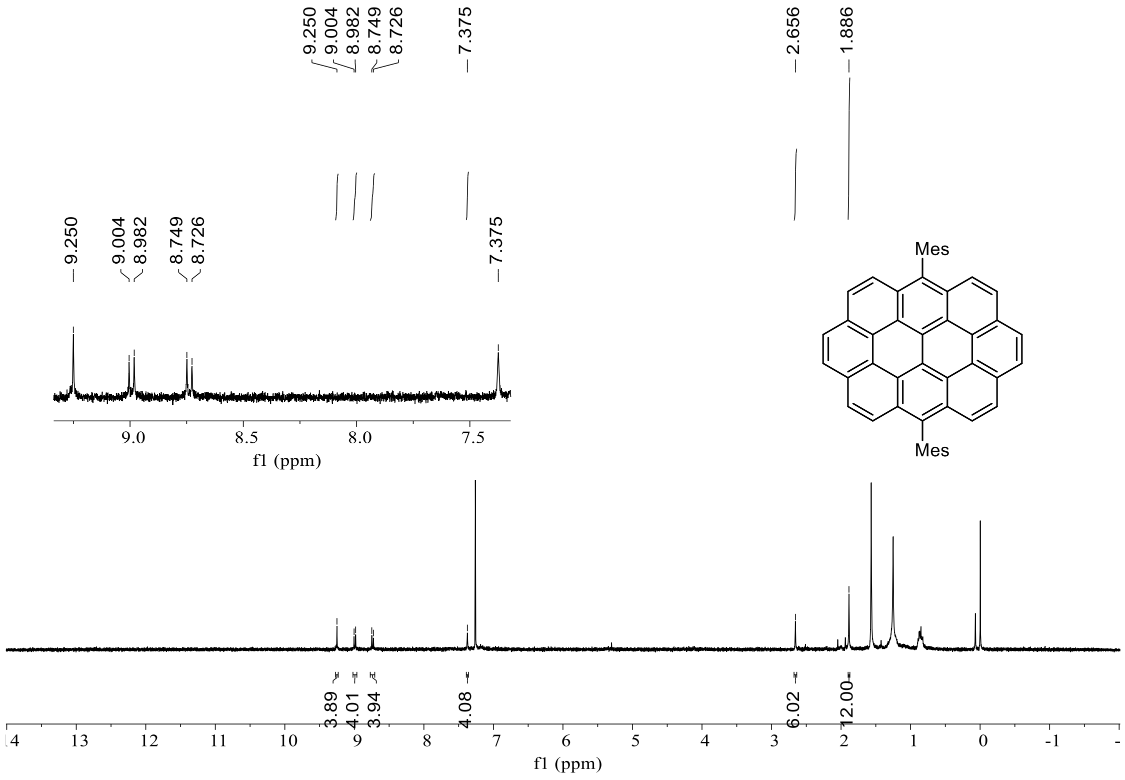

Figure S19. ^{1}H NMR spectra of **1** (400 MHz, $CDCl_3$)

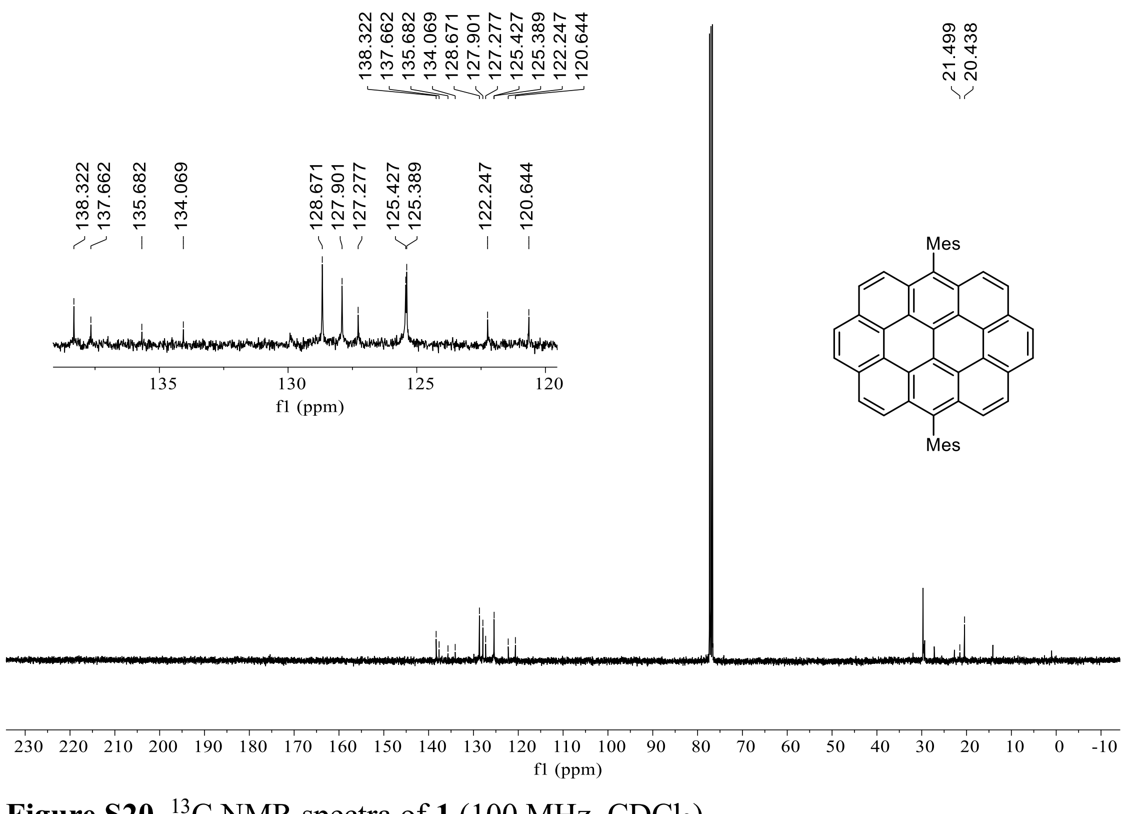

Figure S20. ^{13}C NMR spectra of **1** (100 MHz, $CDCl_3$)

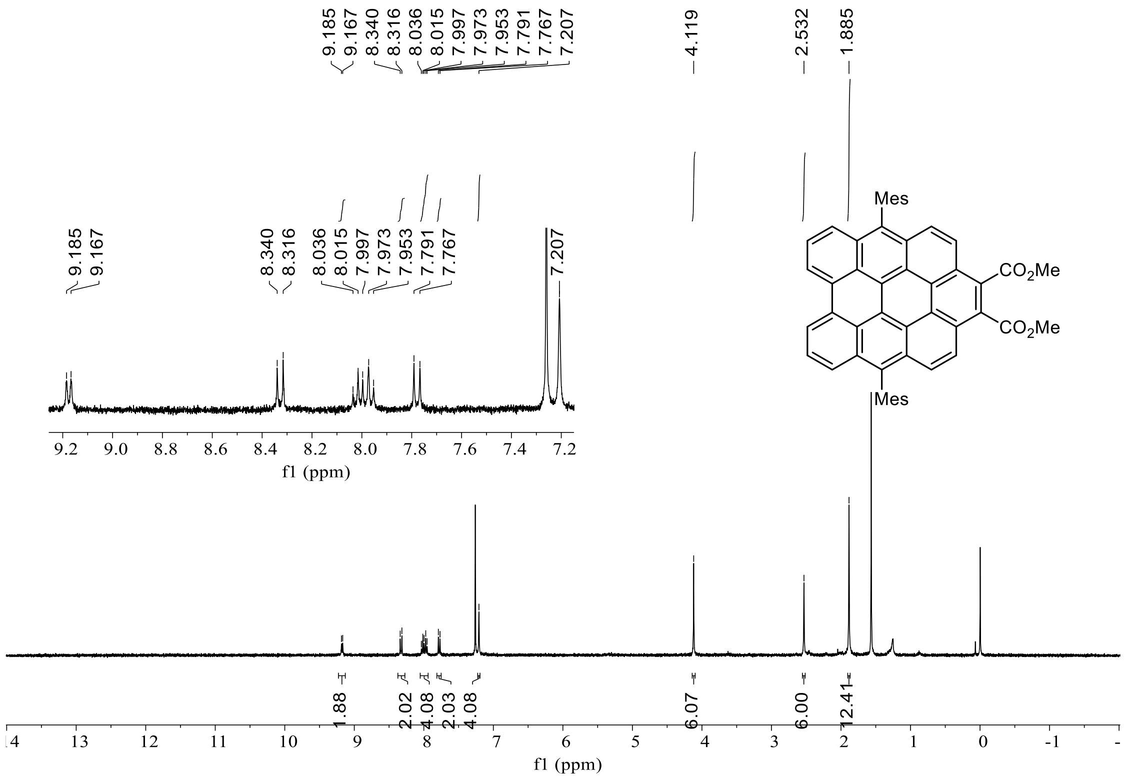

Figure S21. ^{1}H NMR spectra of **S2** (400 MHz, $CDCl_3$)

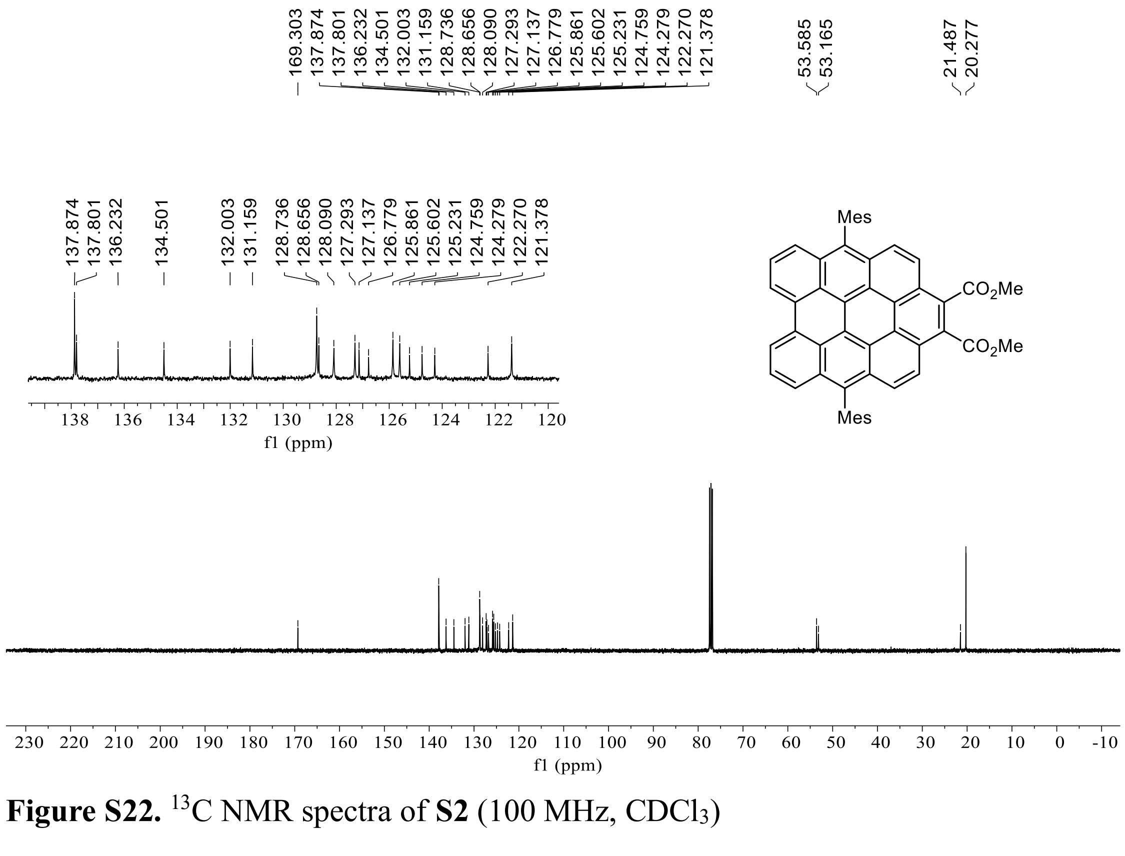

Figure S22. ^{13}C NMR spectra of **S2** (100 MHz, $CDCl_3$)

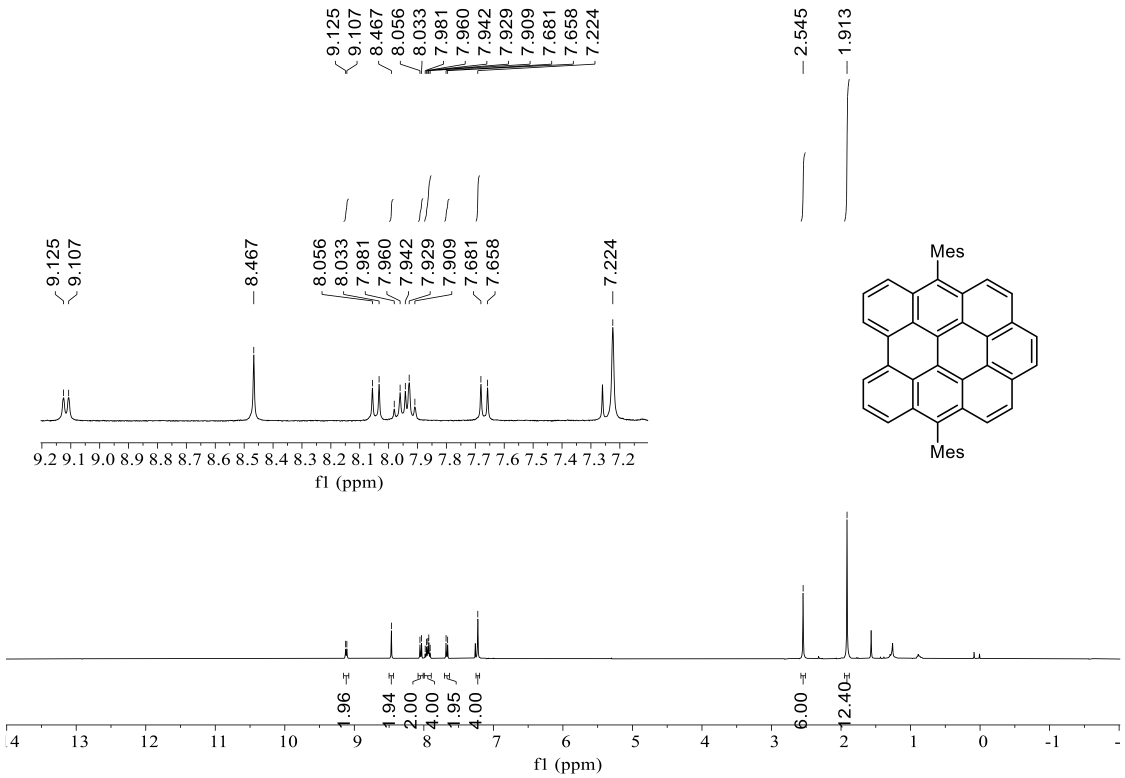

Figure S23. ^{1}H NMR spectra of **3** (400 MHz, $CDCl_3$)

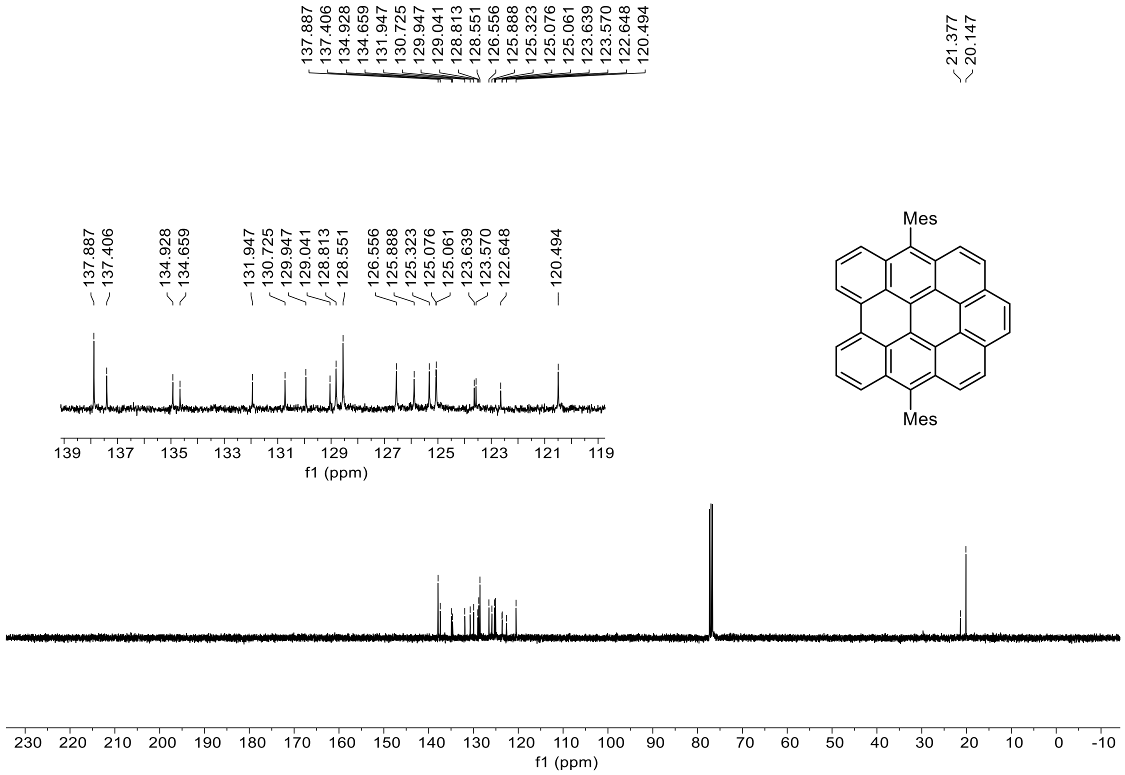

Figure S24. ^{13}C NMR spectra of **3** (100 MHz, $CDCl_3$)

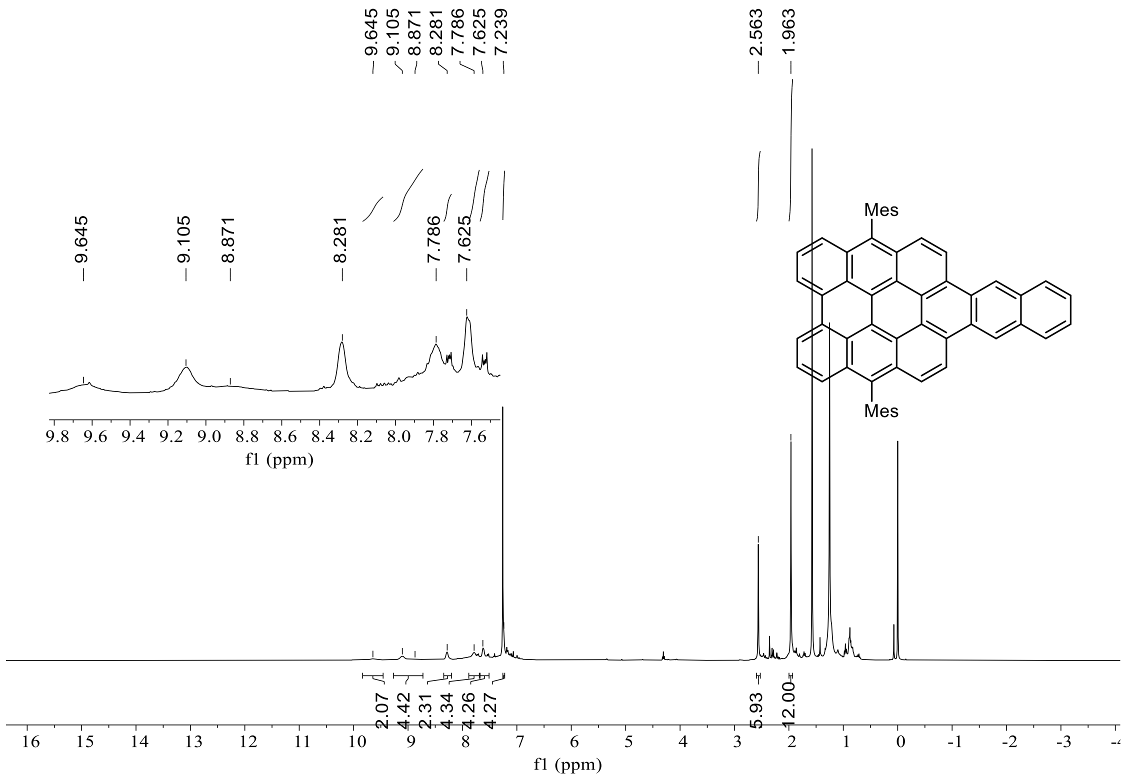

Figure S25. ^{1}H NMR spectra of **4** (400 MHz, $CDCl_3$)

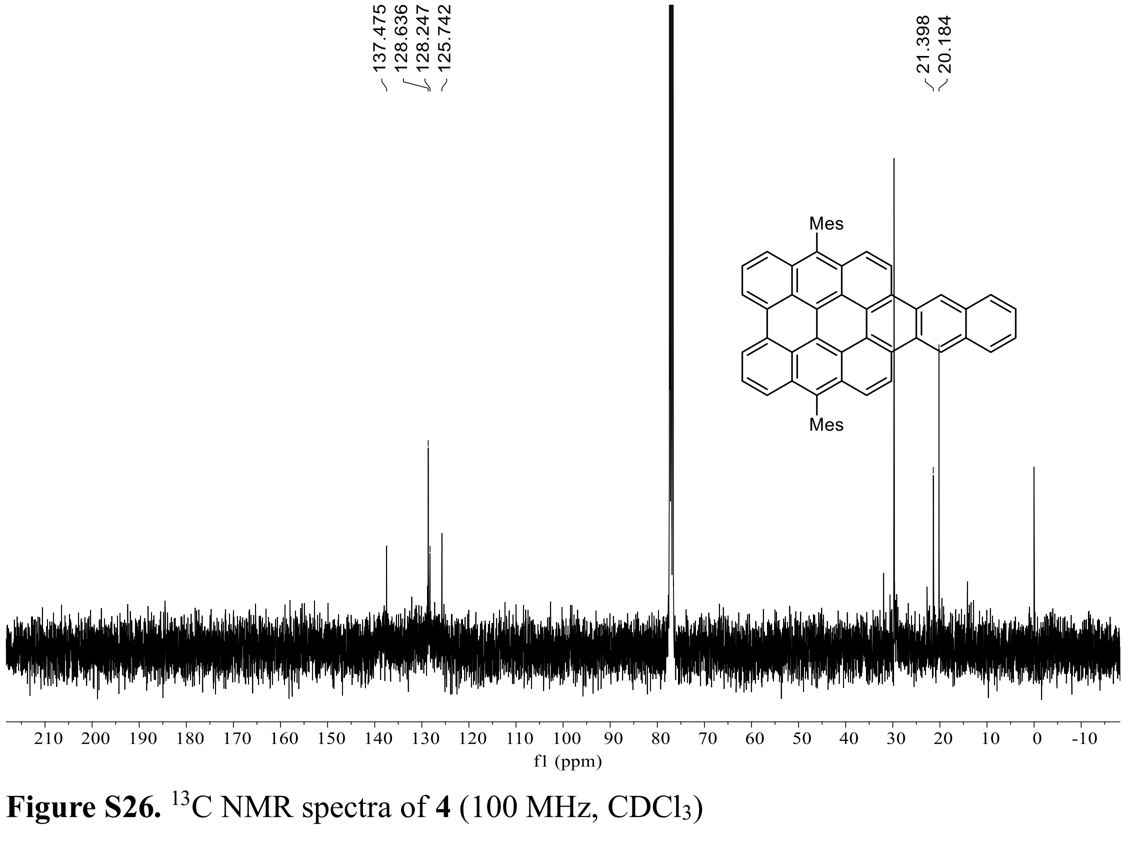

Figure S26. ^{13}C NMR spectra of **4** (100 MHz, $CDCl_3$)

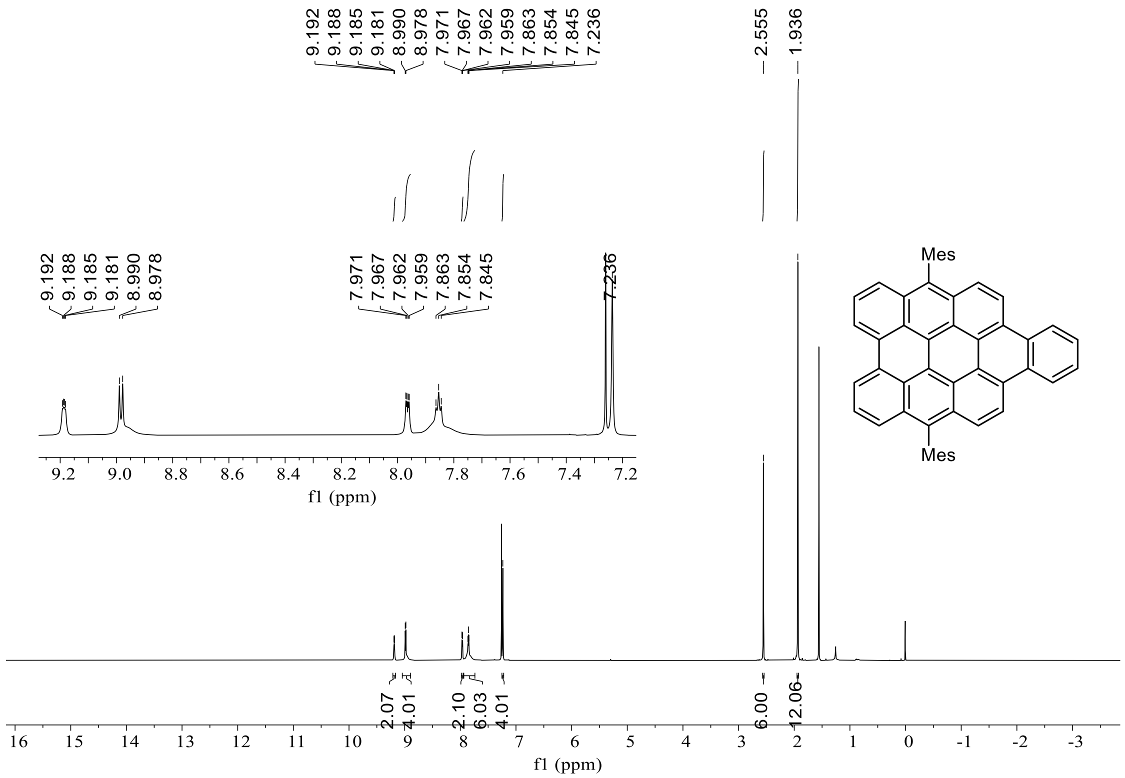

Figure S27. ^{1}H NMR spectrum of **2** ($CDCl_3$, 800 MHz)

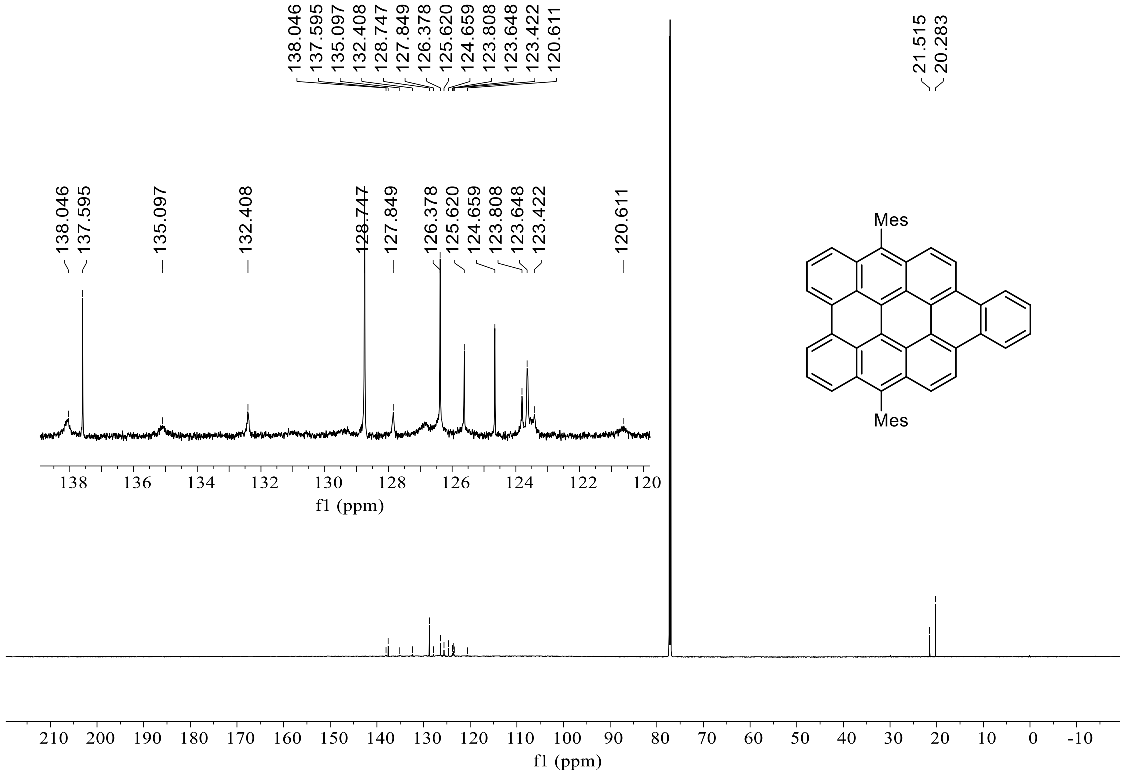

Figure 28. ^{13}C NMR spectrum of **2** ($CDCl_3$, 200 MHz)

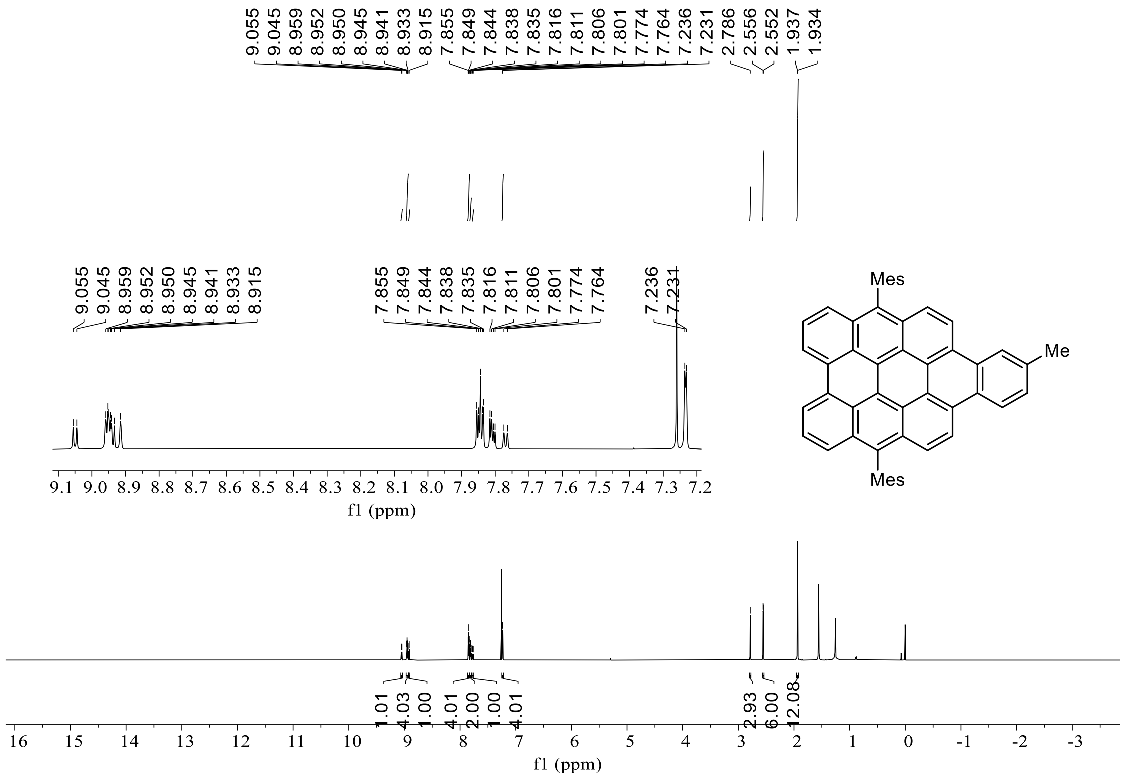

Figure S29. ^{1}H NMR spectrum of **5** ($CDCl_3$, 800 MHz)

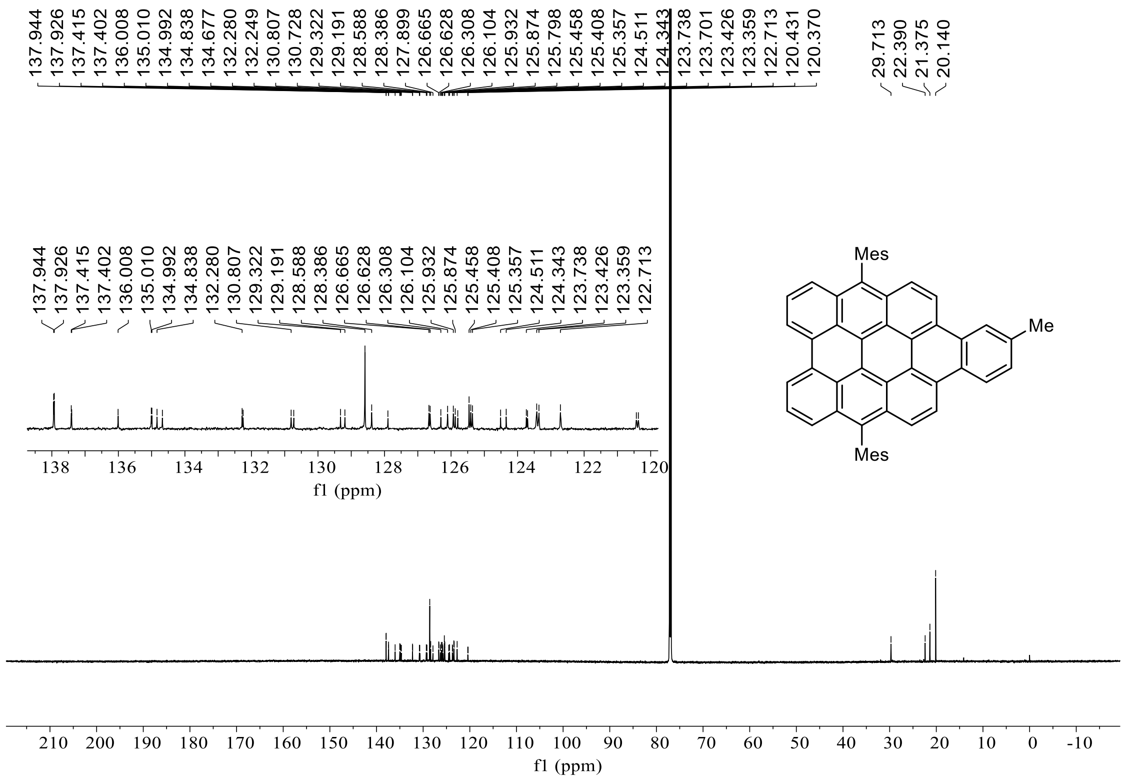

Figure S30. ^{13}C NMR spectrum of **5** ($CDCl_3$, 200 MHz)

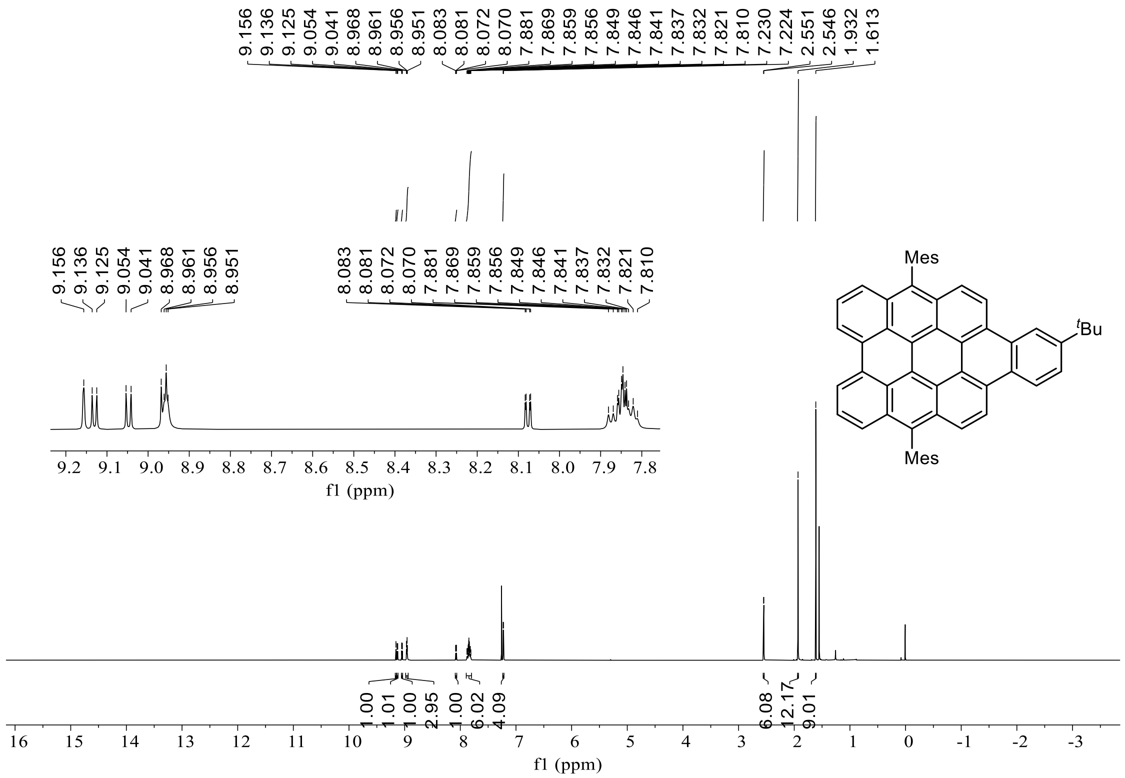

Figure S31. ^{1}H NMR spectrum of **6** ($CDCl_3$, 800 MHz)

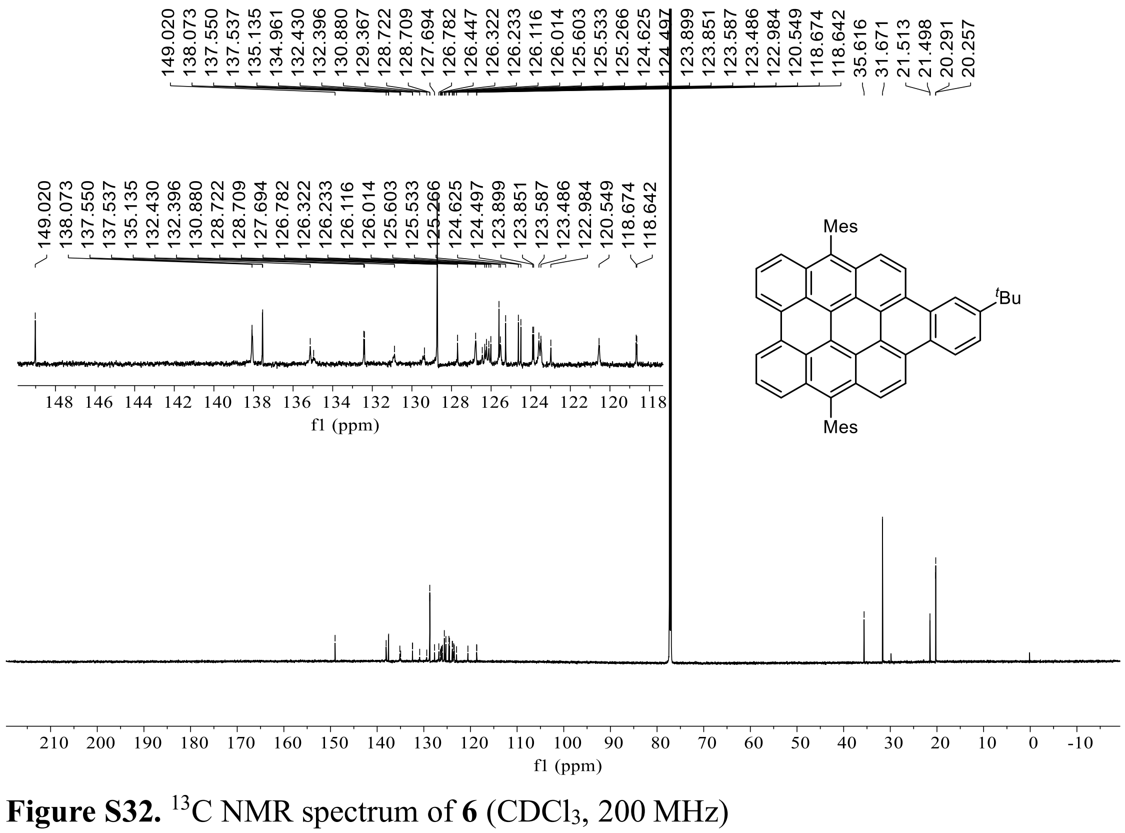

Figure S32. ^{13}C NMR spectrum of **6** ($CDCl_3$, 200 MHz)

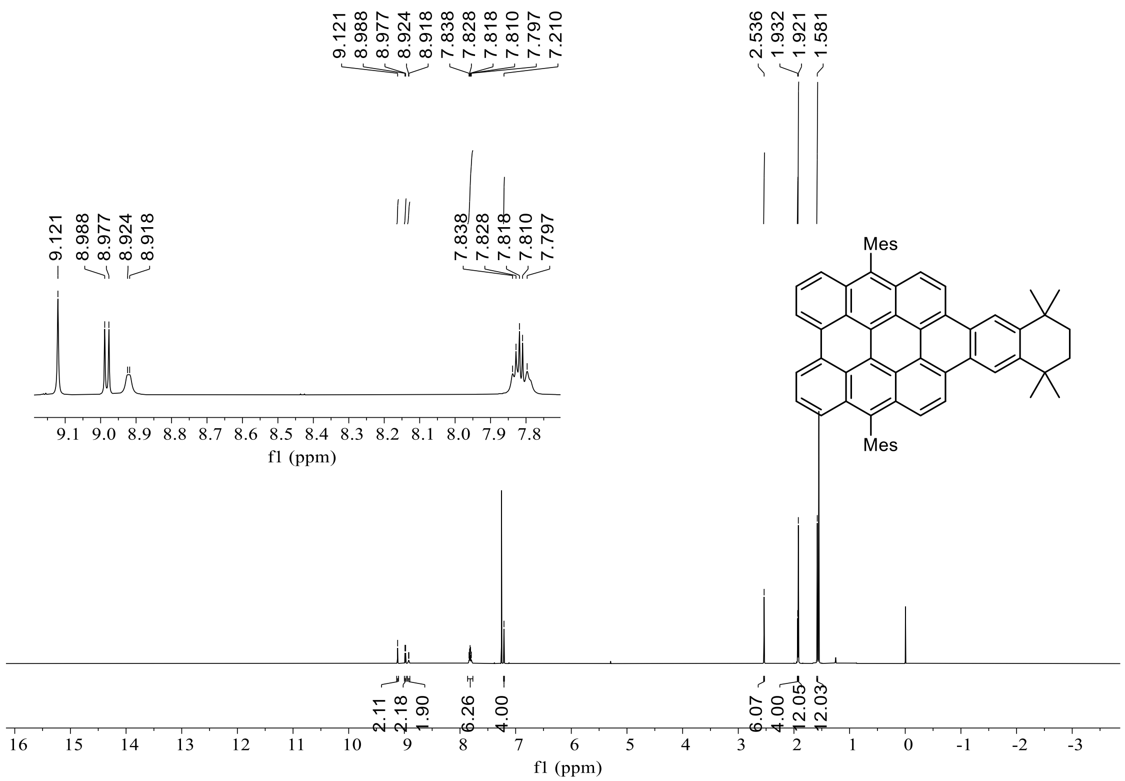

Figure S33. ^{1}H NMR spectrum of **7** ($CDCl_3$, 800 MHz)

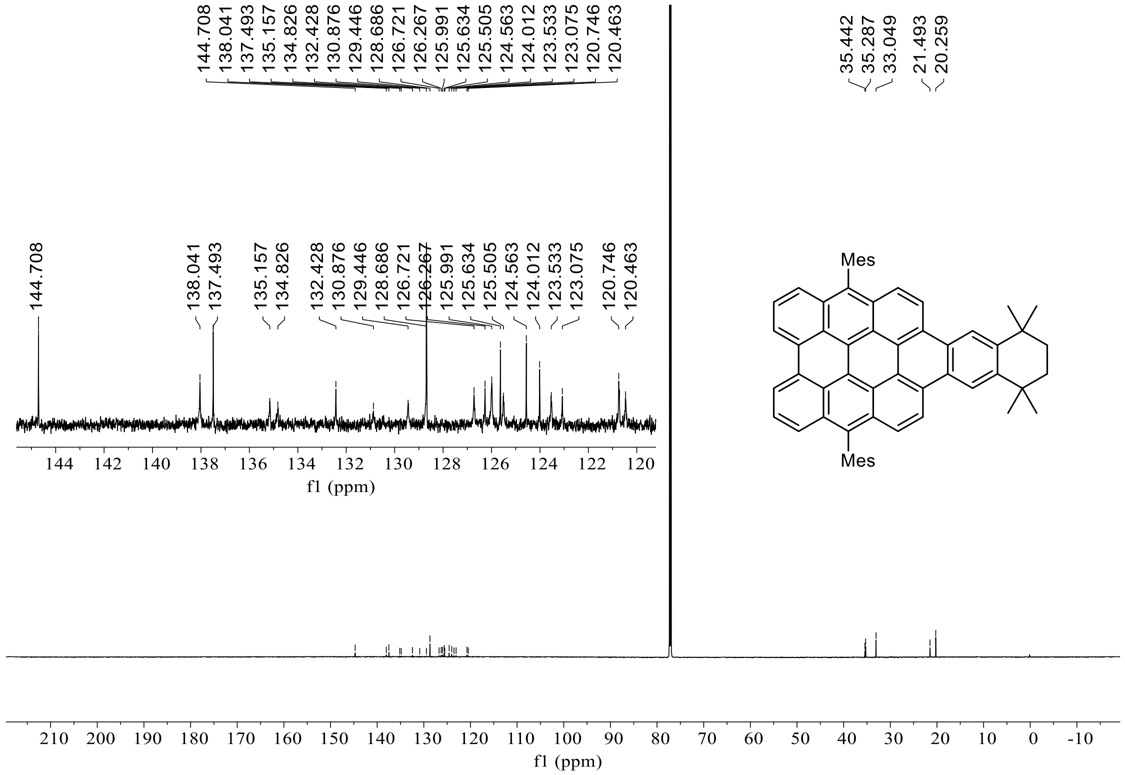

Figure S34. ^{13}C NMR spectrum of **7** ($CDCl_3$, 200 MHz)

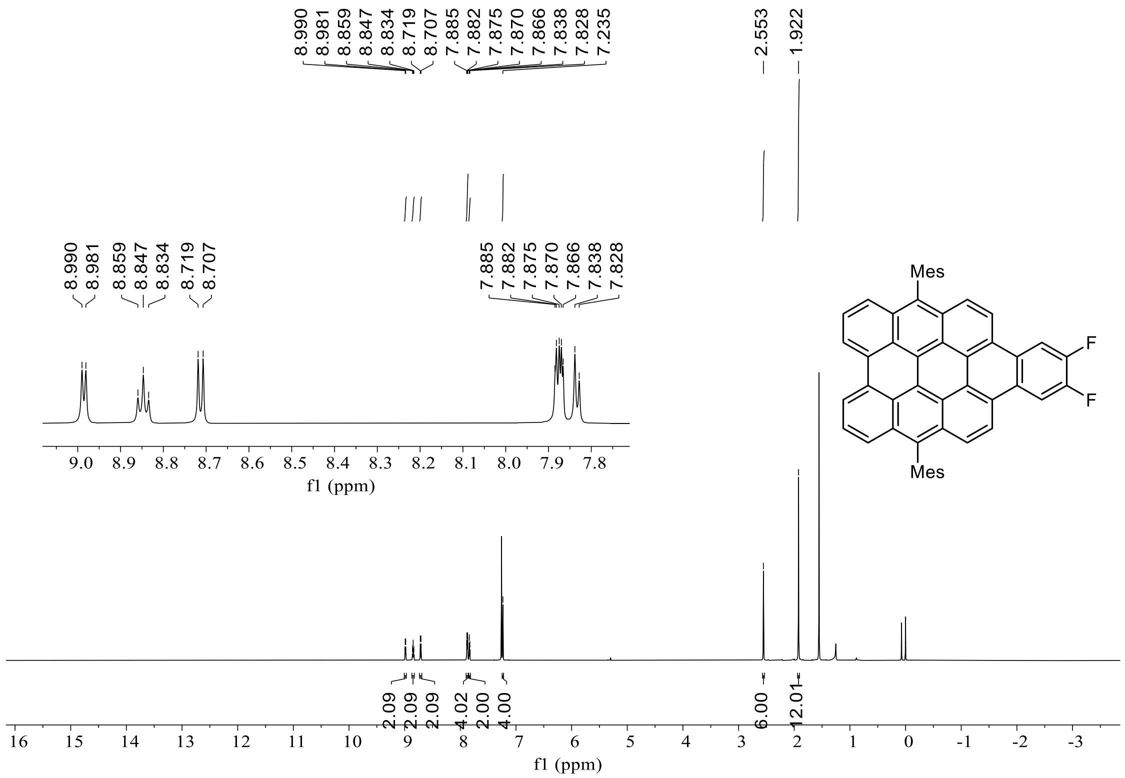

Figure S35. ^{1}H NMR spectrum of **8** ($CDCl_3$, 800 MHz)

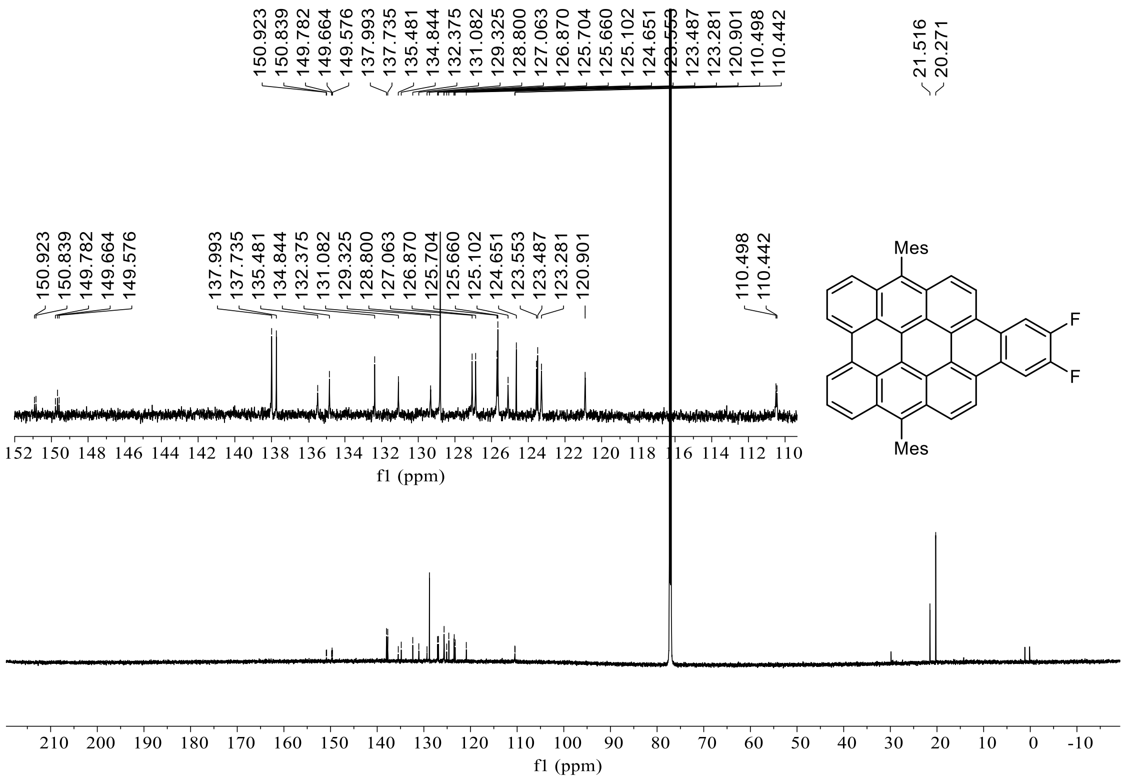

Figure S36. ^{13}C NMR spectrum of **8** ($CDCl_3$, 200 MHz)

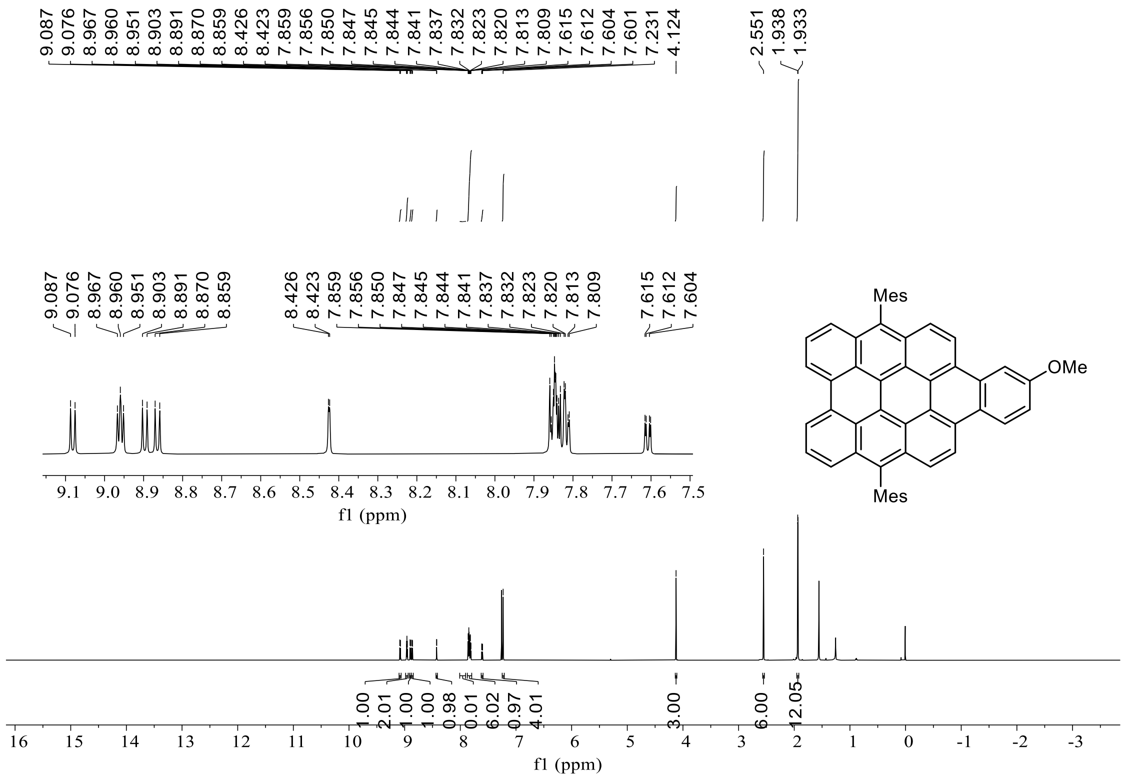

Figure S37. ^{1}H NMR spectrum of **9** ($CDCl_3$, 800 MHz)

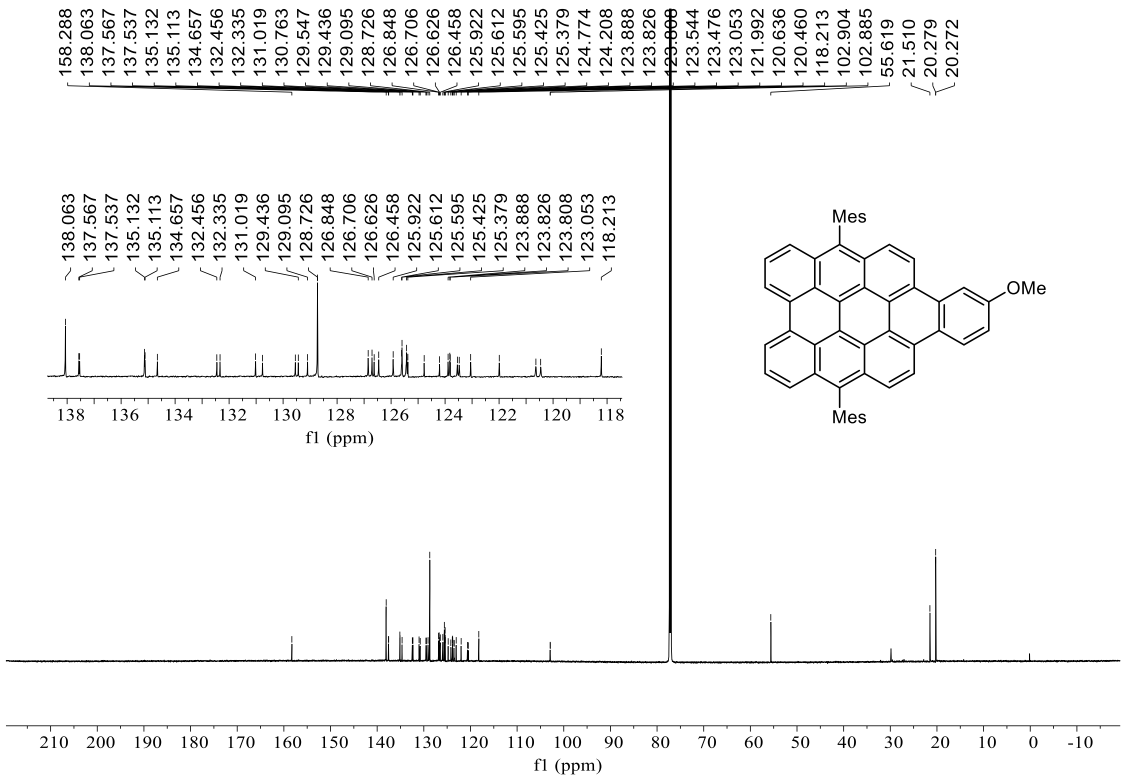

Figure S38. ^{13}C NMR spectrum of **9** ($CDCl_3$, 200 MHz)

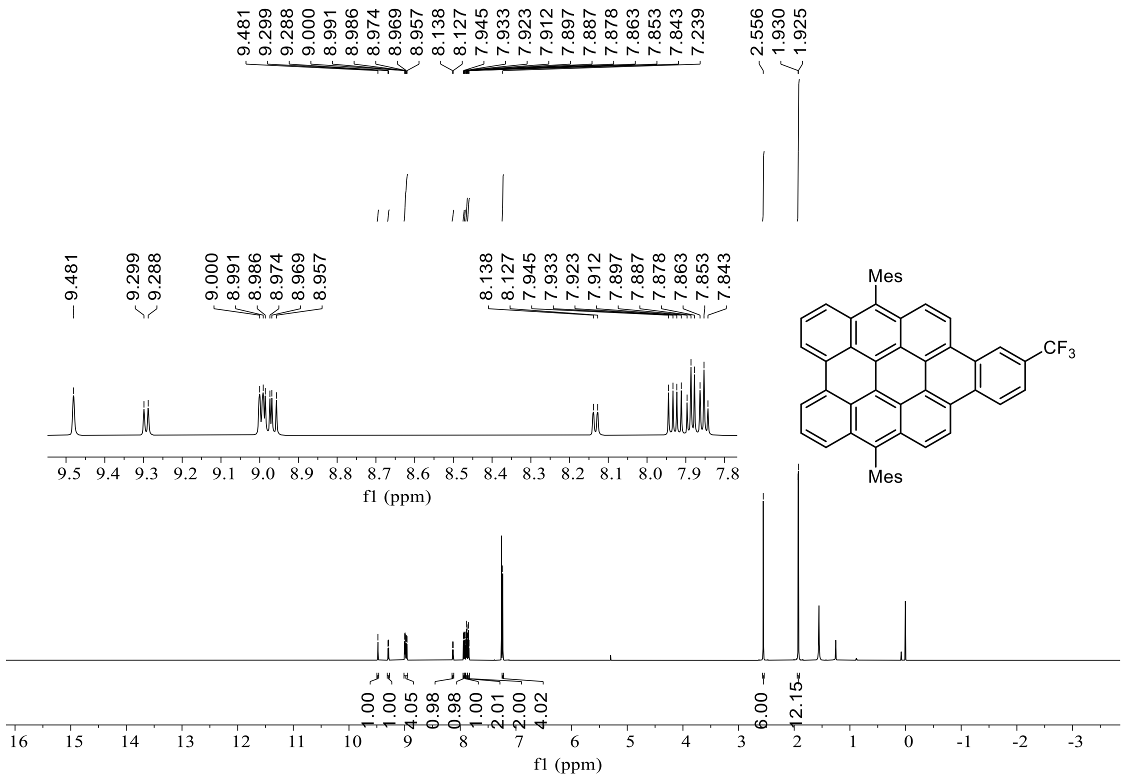

Figure S39. ^{1}H NMR spectrum of **10** ($CDCl_3$, 800 MHz)

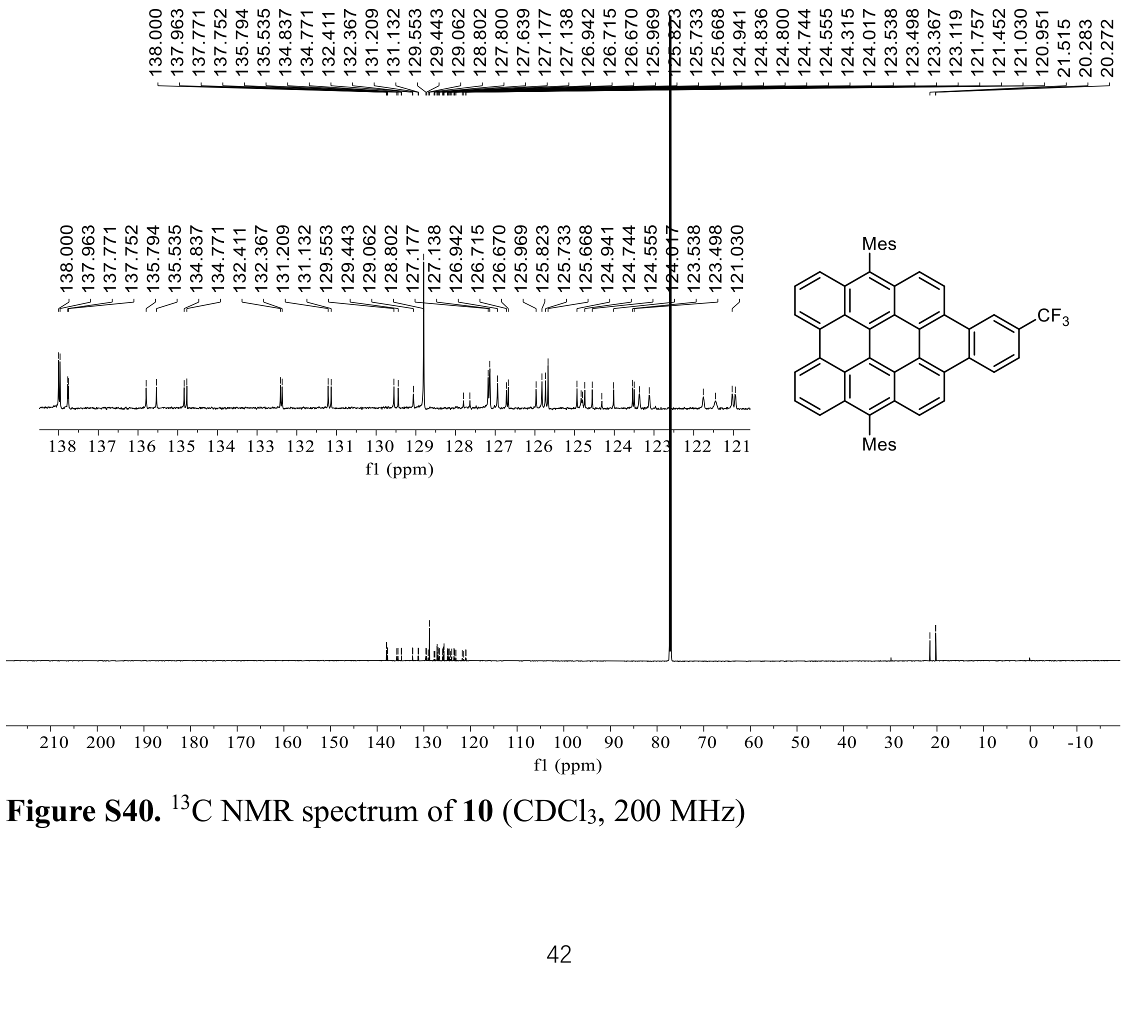

Figure S40. ^{13}C NMR spectrum of **10** ($CDCl_3$, 200 MHz)

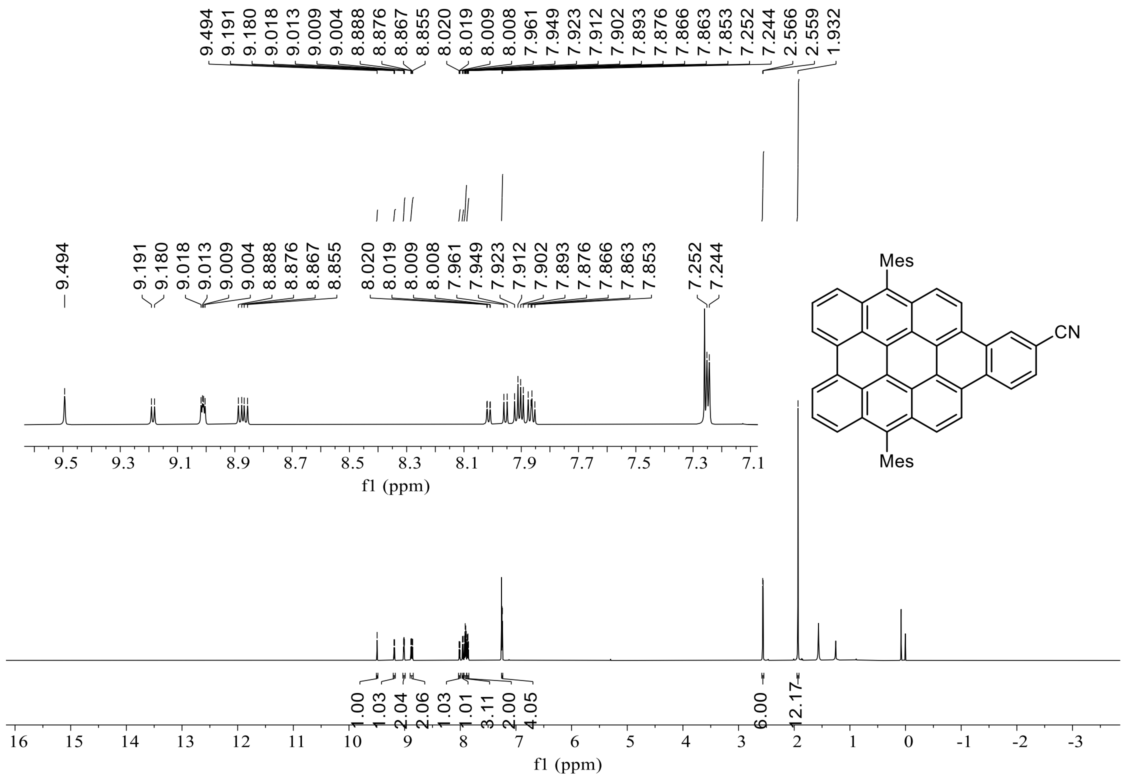

Figure S41. ^{1}H NMR spectrum of **11** ($CDCl_3$, 800 MHz)

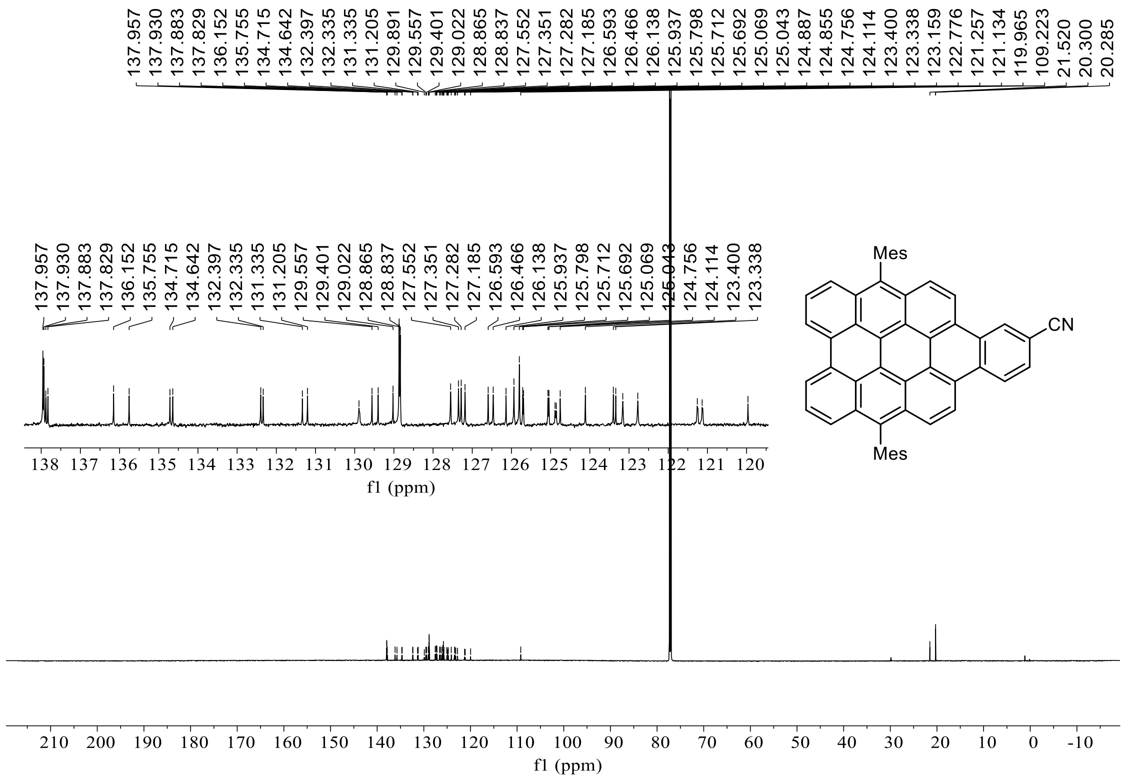

Figure S42. ^{13}C NMR spectrum of **11** ($CDCl_3$, 800 MHz)
